# Scientometric Analysis: An Emerging Tool in Veterinary and Animal Scientific Research

**DOI:** 10.3390/ani14213132

**Published:** 2024-10-31

**Authors:** Georgia A. Vaitsi, Maria V. Bourganou, Daphne T. Lianou, Yiannis Kiouvrekis, Charalambia C. Michael, Dimitris A. Gougoulis, George C. Fthenakis

**Affiliations:** 1 Veterinary Faculty, University of Thessaly, 43100 Karditsa, Greece; gvaitsi@vet.uth.gr (G.A.V.); dgoug@uth.gr (D.A.G.); 2 Faculty of Public and One Health, University of Thessaly, 43100 Karditsa, Greecekiouvrekis.y@uth.gr (Y.K.); 3 School of Veterinary Medicine, European University of Cyprus, Egkomi, Nicosia 2404, Cyprus

**Keywords:** animal science, bibliometric, bibliometric analysis, health sciences, life sciences, meta-analysis, One Health, review, scientometric, veterinary science

## Abstract

The present study is an evaluation of published papers in the field of scientometrics–bibliometrics in veterinary or animal studies. The objective was the quantitative evaluation of the scientific content and the bibliometric details of papers that had used scientometrics as a methodological approach to assess various topics within the broad field of veterinary or animal studies. For the search of published papers, the Web of Science database was used; the search string {ALL FIELDS = [scientometric* OR bibliometric*]} AND {ALL FIELDS = [veterinary OR animal*]} was employed. In all, 163 papers were evaluated individually. There was a progressive increase in the number of papers published annually (slope 0.314 ± 0.063) which originated from 58 countries, mostly from the United States of America (*n* = 24) and 306 organizations. The databases employed most frequently for record search were the Web of Science, Scopus and PubMed (in 105, 60 and 21 papers, respectively); the median timespan of the searches was 25 years (interquartile range (IQR): 31) and the median number of records was 1082.5 (IQR: 4708) (min.: 14, max.: 207,894). The papers were published in 106 journals (*Animals* (*n* = 21), *Scientometrics* (*n* = 8)), classified into 56 Web of Science categories (‘veterinary sciences’ (*n* = 26 journals)). Searches related to 88 different topics. There were 517 unique keywords in the papers, among which ‘bibliometric(s)’ (*n* = 45) occurred most often. The median number of co-authors per paper was 4 (IQR: 3) (min.: 1, max.: 17), with a total of 689 individual authors. Most papers (*n* = 110 (67.5%)) were published under open access. The median number of citations received per paper was 4 (IQR: 9) (max.: 68) and the *h*-index was 21. In multivariable analysis, the higher number of cited references in papers and the inclusion of specific animal species in the record search emerged as significant for the number of citations received per paper yearly.

## Simple Summary

Scientometrics refer to studies that quantify aspects of scholarly literature. The methodology can be a useful tool for understanding scientific trends and for mapping large relevant data. The present work has focused on the application of scientometric analysis in published research papers in the field of veterinary and animal sciences. The findings have indicated the emergence of using this analysis in works in the primary fields of scientific interest of researchers; moreover, they have provided a guide for future relevant studies.

## 1. Introduction

Scientometric evaluations (which include bibliometric analyses) can be useful in understanding scientific trends, in highlighting emerging topics and in mapping large amounts of relevant data [1]. That way, they can provide support to comprehend the constituents of research, the performance of journals and topics, the collaborations between researchers and thus, consequently, the further exploration of scientific research [1,2]. These studies are important parts of the development of research, as well as in its evaluation [3].

Such analyses are based on the identification of the existing scientific literature within the subject area under assessment and involve, primarily, quantitative assessment on the documents retrieved; however, analysis of qualitative traits in the content can also take place as well [3]. An advantage of scientometric analyses refers to the use of large amounts of data, which had been produced by other researchers (e.g., author groups, journals where papers were published, citations of papers), unrelated to the researchers performing the scientometric analyses [4], which leads to reliable and unbiased findings. Further, the increase in scientific output can also contribute to a risk of the traditional-style narrative reviews being potentially incomplete [5]; in this respect, it is hence notable that the availability of scientometric analyses can help people working on such reviews to provide guidance on the trends and significance of research in the topic under study.

A recent (August 2024) search into the Web of Science database by using the string [scientometric* OR bibliometric*] revealed that only 0.21% of retrieved papers were classified into the category ‘veterinary sciences’ and 0.17% into the category ‘agriculture, dairy and animal science’. There were, however, another 33 categories in the database which included ≥1% of total papers retrieved in the search: these included categories in the health sciences (e.g., medicine general internal, surgery) and the life sciences (e.g., environmental sciences). The findings point out that scientometric analyses have not been used extensively in works related to veterinary and animal studies. There is, therefore, interest in further developing the approach in those areas.

The present study is an evaluation of published papers in the field of scientometrics–bibliometrics in veterinary or animal studies. The objective of the study was the quantitative evaluation of the scientific content and the bibliometric details of papers that had used scientometrics as a methodological approach to assess various topics within the broad field of veterinary or animal studies.

## 2. Materials and Methods

### 2.1. Search Procedure

The Web of Science platform (www.webofknowledge.com (accessed on 18 August 2024 and 12 September 2024); Clarivate Analytics, London, UK) was used for the search of relevant publications. For the search, we used the Web of Science Core Collection, in a search that spanned across multiple disciplines, through the inclusion of the Science Citation Index Expanded, the Emerging Sources Citation Index, the Social Sciences Citation Index, the Arts and Humanities Citation Index, the Conference Proceedings Citation Index and the Book Citation Index.

A search using the following string was performed: {ALL FIELDS = [scientometric* OR bibliometric*]} AND {ALL FIELDS = [veterinary OR animal*]} (the asterisk served as a truncation symbol to include variations of the respective terms). The search was performed on 18 August 2024 (‘freeze date’) and was repeated on 12 September 2024 to confirm that no additional records (i.e., delayed entries) had been added thereafter. Only records published up to 30 June 2024 were included in this study.

Thereafter, an initial document type analysis of the records obtained was performed, during which only the following types of documents were included: ‘article’ and ‘review article’. Thus, 759 papers were retained for further assessment individually (Table S1).

### 2.2. Paper Evaluation

Evaluation of characteristics in the papers was performed by two persons. The evaluators worked separately and independently and compared their results after completion of their work. In case of disagreement between the two persons, the paper was evaluated by a senior author (G.C.F.) and the results returned by two of the three assessors were taken into account; this occurred with four papers in total.

During evaluation, papers not including (a) work related to veterinary or animal studies or (b) work related to scientometric or bibliometric assessment were excluded from further evaluation.

Thus, after the above, 163 papers remained and were included in the final scientometric evaluation (Table S1). In each of these papers, the following details were recorded:

1. Year of publication of paper;
2. Country and organization (university or other institution) of origin of the paper (as indicated in the affiliation(s) of the author(s));
3. Database used, timespan set and language employed for the search of records;
4. No. of records assessed in the study;
5. Journal in which the paper was published and Web of Science category(ies) into which each journal was classified;
6. Scientific topic of the scientometric assessment and keywords in the paper;
7. Number of cited references included in the relevant list;
8. Number and names of all co-authors in the paper;
9. Accessibility of paper, i.e., whether it was open access or subscription only;
10. Total number of citations received by the paper until the end of June 2024.

### 2.3. Data Management and Analysis

The number of papers published by 30 June 2024 on [scientometric* OR bibliometric*] only was obtained using the same procedure, in order to compare with the number of papers published on the topic of the current study.

Papers with authors affiliated with organizations of two different countries were deemed as with an international collaboration.

To assess the impact of the papers evaluated in the current study, the total number of citations received by the papers was considered. The number of citations received by the papers was normalized by calculating the average citations per year since the year of publication of each paper.

All data were systematically recorded and organized using Microsoft Excel (versions 2406-2408). Descriptive analysis was performed initially. The frequency of the various outcomes was evaluated in tables of cross-categorized frequency data using the Pearson chi-square test or the Fisher exact test, as appropriate. Comparisons between continuous data were performed using the Mann–Whitney test or the Kruskal–Wallis test. Linear regression analysis was used to establish associations with the year of publication of each paper. Spearman’s rank correlation analysis was performed as indicated, and the significance of the result was evaluated according to the critical values for *r*.

The outcome ‘citations received by a published paper yearly’ was evaluated. Initially, univariable analysis was performed to assess potential associations with relevant parameters. Then, a multivariable model was developed for the above outcome, and parameters found with *p* < 0.20 in the univariable analyses previously carried out were included into this model. Progressively, parameters were removed from the model by using backward elimination. The likelihood ratio test was performed to assess the *p*-value of each parameter; among those found with *p* > 0.20, the one with the largest *p* was removed from the model. The procedure was repeated until no variable with *p* > 0.20 could be removed from the model. The parameters included in the final multivariable assessment are in Table S2. Subsequently, associations of the number of citations received by a published paper yearly and the parameters into that final multivariable assessment were evaluated by principal component analysis.

Statistical significance was defined at *p* < 0.05.

## 3. Results

All 163 individually assessed papers have been indexed in the Web of Science, fulfilled the search criteria and presented scientometric analysis work on veterinary or animal studies (Table S3).

### 3.1. Year of Publication

**Figure 1:**
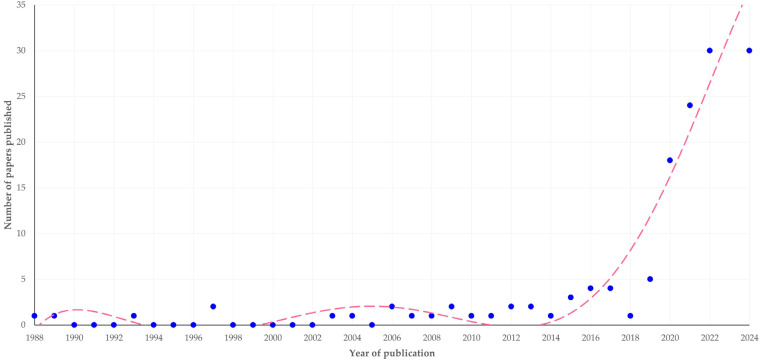
The number of published papers on veterinary or animal studies scientometrics according to the year of publication (the dashed line is trendline).

There was a clear progressive increase in the number of papers published (slope 0.314 ± 0.063; *p* < 0.0001) (Figure 1). The first relevant paper was published in 1988; however, most papers (66.3%) were published during the current decade (2021–today). A similar trend was observed in the entirety of published papers on scientometrics–bibliometrics internationally, where also the majority of papers was published subsequently to 2020 (60.6% of 30,702 papers) (*p* = 0.14) (Figure S1).

### 3.2. Origin of Published Papers

#### 3.2.1. Countries

**Figure 2:**
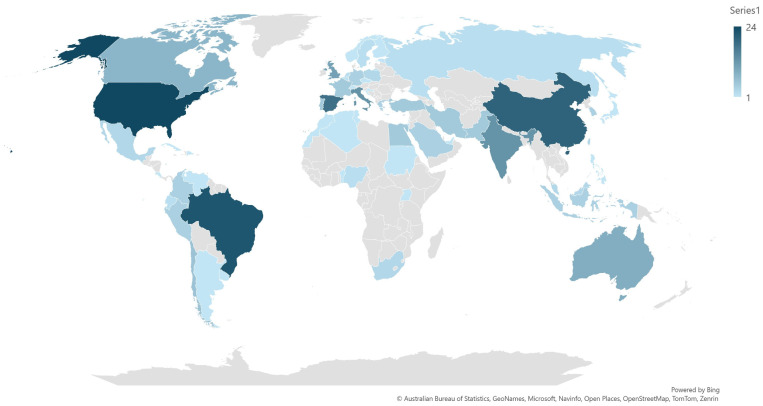
The countries of the world from which published papers on veterinary or animal studies scientometrics originated (blue color palette in accord with the number of papers published per country).

The papers originated from 58 countries in total. The median number of published papers per country that contributed publications was 2 (interquartile range (IQR): 4). Most papers originated from the United States of America (*n* = 24), Brazil (*n* = 22) or China (*n* = 20) (Figure 2, Table S4). Papers published up to the end of 2020 originated from 23 countries, whilst those published afterwards originated from 53 countries (*p* < 0.0002).

**Figure 3:**
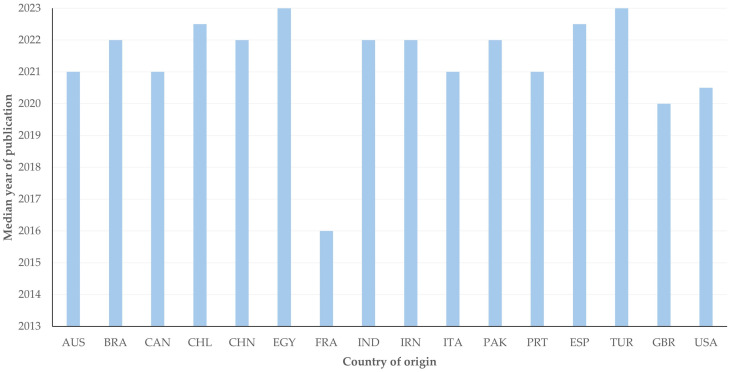
The median year of publication of papers on veterinary or animal studies scientometrics from the 16 countries with most papers (≥5) (the country name abbreviations are set according to International Naming Convention ISO 3166 [6]).

There were 16 countries, from which at least five published papers originated. There was a significant difference in the median year of publication of papers among these countries; France had the oldest median year of publication (2016 (IQR: 1)), whilst Egypt and Turkey had the most recent median year of publication (2023 (IQR: 1)) of relevant papers (*p* = 0.020) (Figure 3).

In 57 papers (35.0%) there was international collaboration (i.e., with affiliations from at least two countries). International collaborations were seen in 52 countries, and, in contrast, 6 countries published papers on their own only. The most frequent combination in joint papers was between Spain and the United States of America (*n* = 4) (Table S5).

#### 3.2.2. Organizations

The papers originated from 306 organizations in total. These included 240 universities and 66 organizations of other type (e.g., research institutes, government agents). The median number of published papers per organization was 1 (IQR: 0).

**Figure 4:**
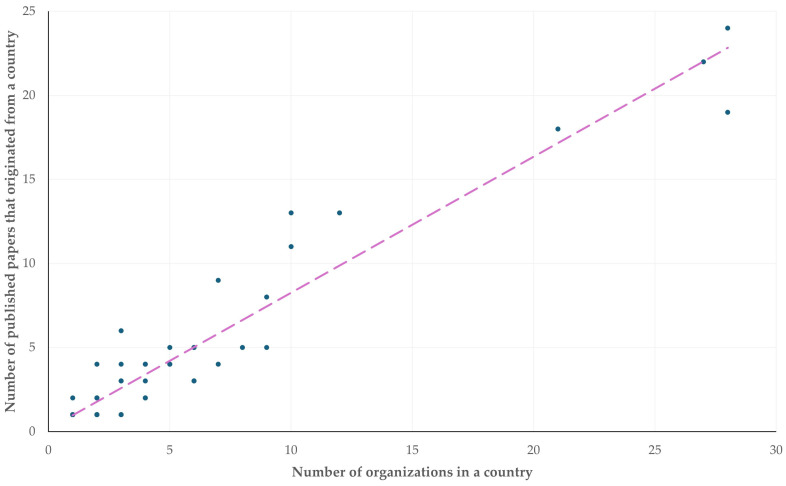
The number of organizations in a country, from which published papers on veterinary or animal studies scientometrics originated, and the number of published papers from that country (the dashed line is trendline).

Most papers originated from the North Carolina State University and the São Paulo State University (*n* = 5) (with reference to universities), and from the Indian Council of Agricultural Research (*n* = 7) (Table S6). China and the United States of America were the two countries with the most organizations from which relevant papers originated (*n* = 28 for both). There was a clear correlation between the number of organizations in a country from which papers originated, and the number of published papers from that country (*r_sp_* = 0.882, *p* < 0.0001) (Figure 4).

### 3.3. Search Methodologies

#### 3.3.1. Databases Used for Search of Records

**Figure 5:**
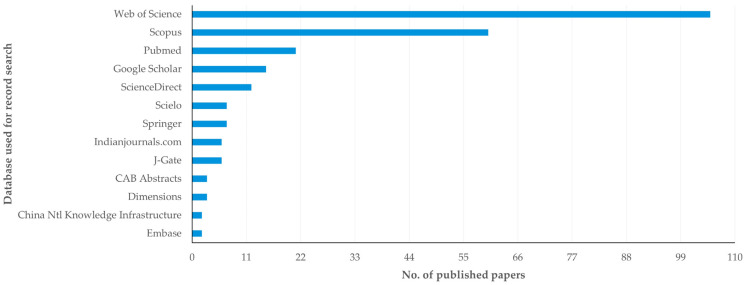
The databases used most frequently for record search in published papers on veterinary or animal studies scientometrics, with the number of published papers in which their use was reported (full details in Table S7).

In total, 40 different databases were employed for search of records in the studies reported in the papers (Table S7). The databases employed most frequently were the Web of Science (in 105 papers), Scopus (in 60 papers) and PubMed (in 21 papers) (Figure 5). In most papers (*n* = 121; 74.2%), use of only one database was reported for search of records, whilst in 42 papers (25.8%) use of at least two databases was reported.

There was no difference in the median year of publication of papers in accord with the database employed (Table S8) (*p* = 0.70). However, there was a clear difference among databases in the countries that used them (*p* = 0.003) (Table S9). Specifically, the Web of Science was employed more frequently in studies from Australia, Iran, Portugal (100%) and China (90.0%); Scopus was employed more frequently in studies from India (76.9%) and Brazil (68.2%); and PubMed in studies from India (53.8%) and USA (25.0%).

#### 3.3.2. Timespan Set in Searches

The median timespan of searches performed in the studies reported in the various papers was 25 years (interquartile range: 31 years; minimum: 1, maximum: 223 years). There was no significant difference in the timespan of searches in accord with the database employed in the respective study (*p* = 0.89) (Figure S2).

#### 3.3.3. Languages Employed for Search of Records

Only 15 papers reported searches performed with language limitations. Of these, in 12 papers, the language specified in the search was English only; in one paper, the language specified in the search was Spanish only; in one paper, two languages were specified in the search (English and Russian); and in another one, eight languages were specified in the search (Czech, English, Japanese, Norwegian, Polish, Russian, Slovak and Swedish).

#### 3.3.4. Number of Records Found

**Figure 6:**
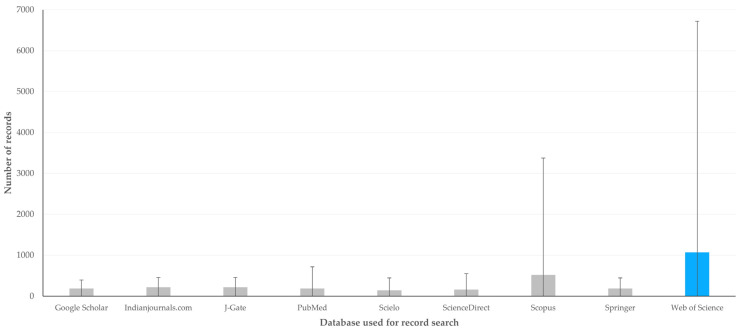
The number of records obtained by the most frequently used databases reported in papers on veterinary or animal studies scientometrics (bars show interquartile range).

The median number of records found in the various studies was 1082.5 (interquartile range: 4708; minimum: 14, maximum: 207,894). There was evidence that in studies which used the Web of Science database, significantly more records were found for use than in studies which used other databases (*p* = 0.0001) (Figure 6, Table S10). In contrast, there was no association between the number of records found in the studies and the timespan covered in these searches (*r_sp_* = 0.086, *p* = 0.09) or the language in which searches were performed (*p* = 0.22). Finally, there was a clear inverse correlation between the number of records found in the studies and the year of publication of the paper (*r_sp_* = –0.334, *p* < 0.0001).

### 3.4. Journals in Which Papers Were Published

**Figure 7:**
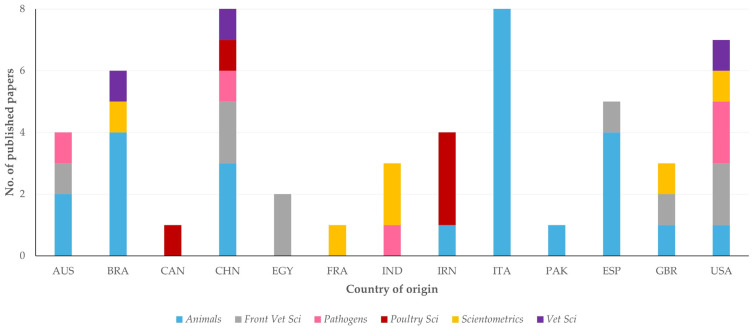
The number of published papers on veterinary or animal studies scientometrics, in accord with the country of origin and the journal of publication (abbreviations of country names according to International Naming Convention ISO 3166 [6]; abbreviations of journals from left to right: *Animals*, *Frontiers in Veterinary Science*, *Pathogens*, *Poultry Science*, *Scientometrics*, *Veterinary Sciences*).

The papers were published in a total of 106 journals, most frequently in *Animals* (*n* = 21 papers), *Scientometrics* (*n* = 8), *Frontiers in Veterinary Science* (*n* = 7) and *Pathogens* (*n* = 4) (Table S11). In total, 24.5% of all papers were published in these four journals, whilst 49.1% of all papers were published in the 23 journals with two or more papers in the study topic (*n* = 80 papers). Cumulatively, 65 of these papers (81.3%) originated from the 16 countries with the most papers published. There were, however, differences among these countries in the journals in which these papers were published (*p* < 0.0001) (Figure 7, Table S12).

These 106 journals were classified into 56 Web of Science categories, most frequently in ‘veterinary sciences’ (*n* = 26 journals, with 61 papers published in these journals), ‘environmental sciences’ (*n* = 13, with 16 papers published in these journals) and ‘agriculture, dairy and animal science’ (*n* = 11, with 36 papers published in these journals) (Table S13). The largest density of published papers per journal within a category was seen in the category ‘computer science, interdisciplinary applications’, with 8 papers in one journal (namely *Scientometrics*), followed by ‘agriculture, dairy & animal science’ (3.3 papers per journal) and ‘microbiology’ (2.5 papers per journal) (Table S13).

**Figure 8:**
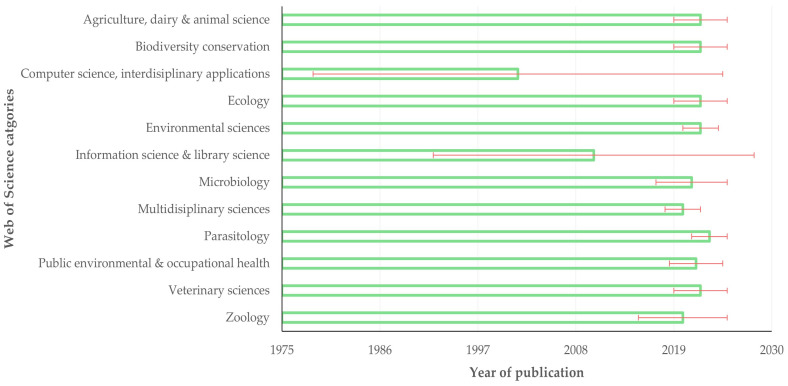
Median year of publication of papers on veterinary or animal studies scientometrics, in accord with Web of Science categories with the most published papers therein (bars indicate interquartile range).

Among the categories with the most published papers in journals therein, ‘parasitology’ (2023 (IQR: 2)); ‘environmental sciences’ (2022 (IQR: 2)); and ‘agriculture, dairy and animal science’, ‘biodiversity conservation’, ‘ecology’ and ‘veterinary sciences’ (2022 (IQR: 3)) included the most recently published papers, whilst the categories ‘computer science, interdisciplinary applications’ (2001.5 (IQR: 23)) and ‘information science and library science’ (2010 (IQR: 18)) included the oldest published papers. The difference between the categories was significant (*p* < 0.0001) (Figure 8).

### 3.5. Content of Papers

#### 3.5.1. Topics of Scientometric Assessment

Searches were found to be related to 88 different topics of scientometric assessment in total. The most frequent search topics referred to country-related research (*n* = 16); the other frequent topics were specific journal-related research, or animal species-related research, or animal welfare (*n* = 10 for each). Details are in Table 1 and Table S14.

**Figure 9:**
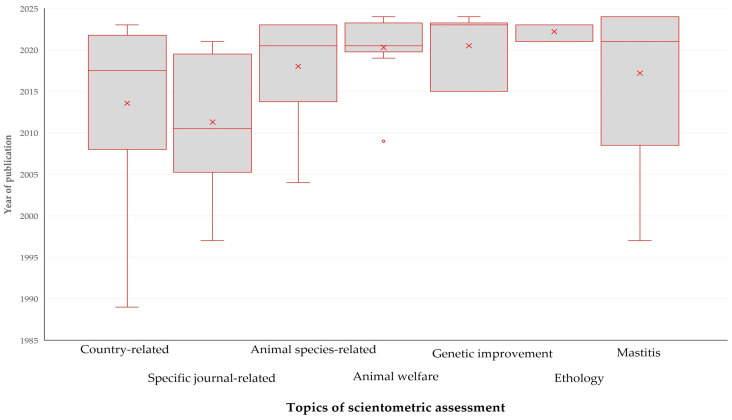
Box and whisker plot of the year of publication in papers on veterinary or animal studies scientometrics in accord with the topic of study and scientometric assessment (only most frequently assessed topics shown).

There was a significant difference in the year of publication of papers in accord with the topic of assessment (*r_sp_* = 0.438, *p* = 0.0004). Papers on scientometrics of genetic improvement or animal ethology were the most recent (median year of publication: 2023); papers on journal-related (median year: 2011) or country-related scientometrics (median year: 2018) were the oldest (Figure 9, Table S18).

In contrast, no association was found between the topic of assessment and the country of origin of papers (*p* = 0.16). Notably, most papers on scientometrics of animal welfare originated from Australia (40.0%), on scientometrics of genetic improvement from Brazil (50.0%) and on scientometrics of mastitis from Greece (40.0%) and the United States of America (40.0%).

Also, no pattern emerged regarding the journals in which the various topics were published. It is, however, noted that most papers on scientometrics of mastitis were published in *Pathogens* (42.9%) and most papers on scientometrics of animal welfare were published in *Animals* (40.0%).

**Figure 10:**
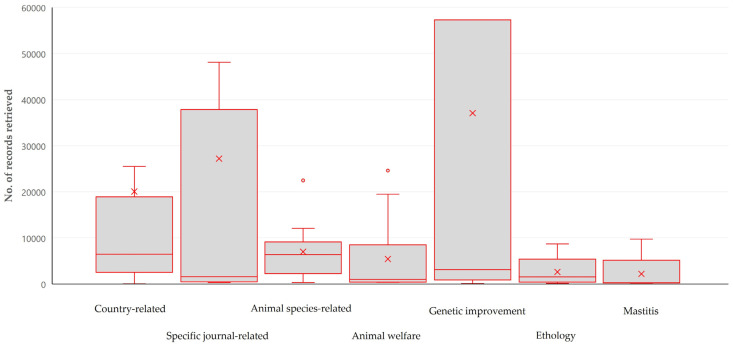
Box and whisker plot of the number of obtained records in papers on veterinary or animal studies scientometrics in accord with the topic of study and scientometric assessment (only most frequently assessed topics shown).

There was an association between the number of records obtained and the topic of scientometric assessment: the median number of records obtained for studies reported in papers on country-related scientometrics (6465 (IQR: 12,954)) or on animal species-related scientometrics (6362 (IQR: 5176)) were the highest, whilst the median number of records for papers on mastitis (250 (IQR: 336)) were the lowest (*r_sp_* = 0.319, *p* = 0.015) (Figure 10, Table S19).

#### 3.5.2. Animal Species Referred to in Scientometric Assessments

In total, 92 papers reported studies with reference to specific animal species in the respective searches. Animal species most frequently studied were cattle (*n* = 14 papers); poultry (*n* = 12 papers); and pigs, sheep and wildlife (*n* = 9 papers each) (Table S20).

Among these papers, there was no association between the animal species referred to and the number of records obtained (*p* = 0.29 when all animal species were taken into account, *p* = 0.09 when only the 10 animal species in most papers were taken into account) (Figure S3).

#### 3.5.3. Keywords in Papers

**Figure 11:**
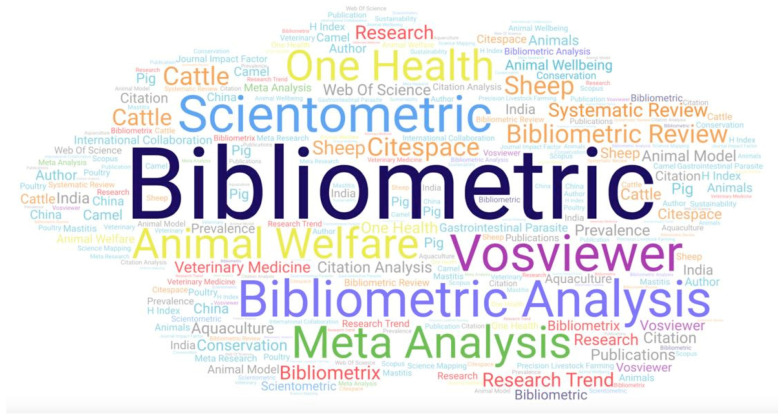
Word cloud of the most frequently occurring keywords in published papers on veterinary or animal studies scientometrics (full details in Table S21).

In total, there were 790 keywords in the 163 papers, i.e., a median number of 5 (IQR: 2) keywords per paper. These corresponded to 517 unique keywords, among which ‘bibliometric(s)’ (*n* = 45), ‘bibliometric analysis(es)’ (*n* = 32) and ‘scientometric(s)’ (*n* = 17) occurred the most frequently (Figure 11, Table S21).

Notably, there was a clear increase in the number of keywords in a published paper in accord with the year of publication (*r_sp_* = 0.275, *p* = 0.0004). Further, there was also a tendency for inverse correlation between the number of keywords in the published papers and the number of records obtained in the search (*r_sp_* = −0.156, *p* = 0.051).

#### 3.5.4. Cited References in Papers

The median number of cited references in the papers was 47 (interquartile range: 44). There was a clear positive correlation between the number of cited references therein and the year of publication of the paper (*r_sp_* = 0.462, *p* < 0.0001), as well as an inverse correlation between the number of cited references therein and the numbers of retrieved records in the paper (*r_sp_* = −0.169, *p* = 0.034).

### 3.6. Authors

**Figure 12:**
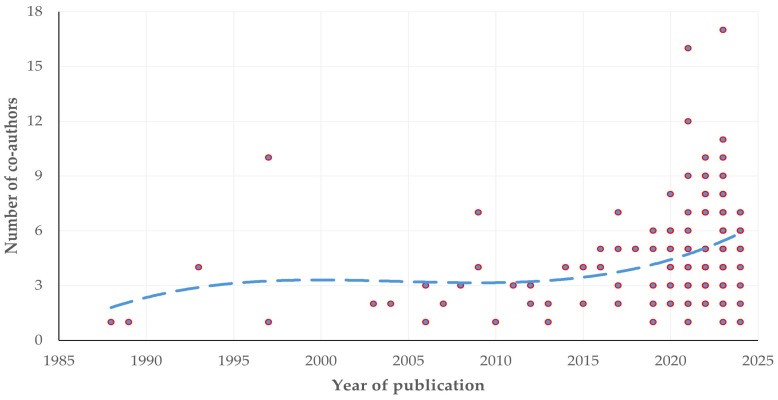
The number of authors per published paper in accord with the year of publication of the paper (the dashed line is trendline).

Cumulatively, there were 770 co-authors; the median number of co-authors per paper was 4 (IQR: 3) (min.: 1, max.: 17). In total, there were 689 individual authors. Among them, 11 (1.6%) had published at least three papers (max.: 5). In 11 papers, there was only one author. There was a clear progressive increase in the number of co-authors per published paper throughout the years (slope: 0.117 ± 0.08) (*p* = 0.0002) (Figure 12 and Figure S4).

Further, there were 286 individual authors (41.5% of all), who were first or last authors in the papers. Among these, one author was first or last in four papers and 26 authors in two papers. The 11 authors with at least three papers, were, on average, first or last authors in 66.7% (25.0%) of their papers (min.: 0.0%, max.: 100.0%), whilst the 52 authors with two papers, were, on average, first or last authors in 50.0% (100.0%) of their papers.

Of the 11 authors with at least three papers as first or last, 9 were affiliated with scientific organizations in the 16 countries from which originated most published papers. Among these 11 authors, there were three clearly distinct groups of co-authors identified with joint papers, who were affiliated with the same organization in each of three different countries, specifically in India (*n* = 5 co-authors), France (*n* = 2 co-authors) and Greece (*n* = 2 co-authors).

There were no significant differences between the 16 countries with most published papers in the median number of authors per paper: papers from Canada had the smallest median number of authors, 3 (IQR: 4.5), whilst papers from Pakistan and Egypt had the highest number, 7 (IQR: 0) and 8 (IQR: 2), respectively (*p* = 0.24) (Figure S5).

The Web of Science categories of the journals of papers published by the above 11 authors, were as follows: (a) agriculture, dairy and animal science, (b) agronomy, (c) computer science, interdisciplinary applications, (d) ecology, (e) infectious diseases, (f) information science and library science, (g) microbiology, (h) parasitology, (i) public environmental and occupational health, (j) veterinary sciences and (k) zoology.

### 3.7. Accessibility of Papers

**Figure 13:**
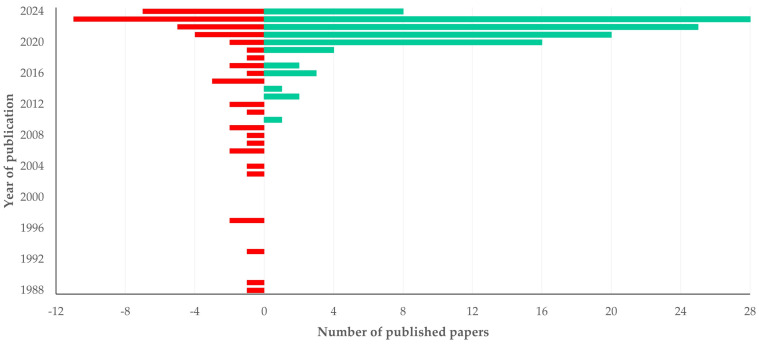
Frequency of published papers on veterinary or animal studies scientometrics, in accord with year of publication and accessibility of papers (red: subscription access, green: open access).

Most papers (*n* = 110 (67.5%)) were published under open access. The median year of publication of open access papers was significantly more recent than that of subscription access papers: 2022 (IQR: 3) versus 2021 (IQR: 12) (*p* = 0.021) (Figure 13). No differences were seen among countries in the proportion of papers published under open access (*p* = 0.22) (Figure S6).

### 3.8. Impact of Papers

The median number of citations received per paper was 4 (IQR: 9) (max.: 68) and the *h*-index for this set of published papers was 21. The median value of yearly citations received per paper was 1.3 (IQR: 2.8) (max.: 21) and the *m*-index was 0.5.

**Figure 14:**
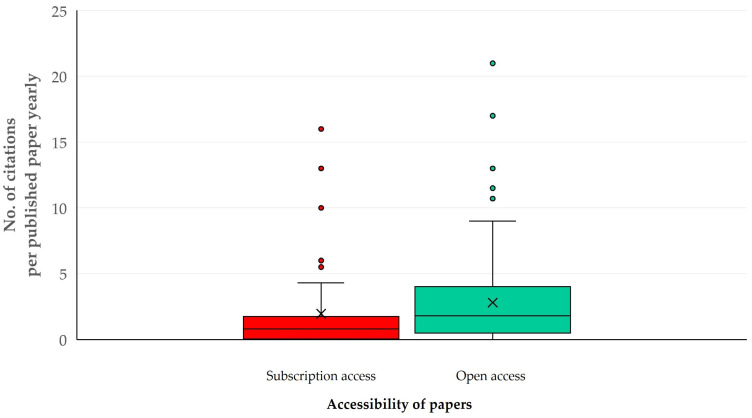
Box and whisker plot of the yearly citations per published paper on veterinary or animal studies scientometrics, in accord with accessibility of papers.

**Figure 15:**
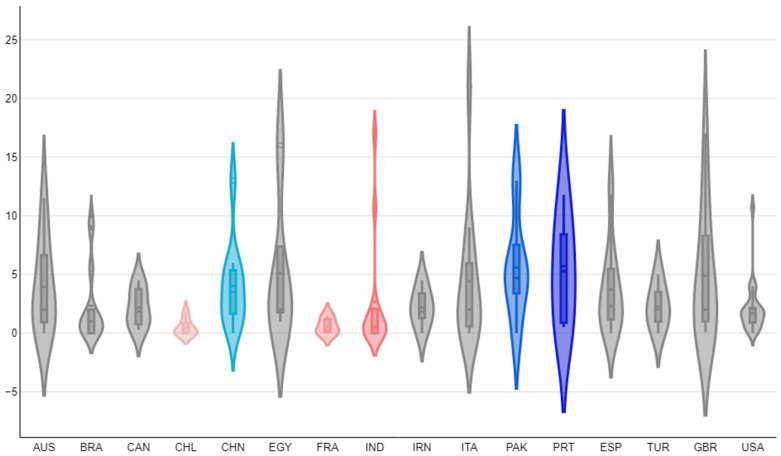
Violin plot of the yearly citations per published paper on veterinary or animal studies scientometrics, among the 16 countries with most relevant published papers (abbreviations of country names according to the International Naming Convention ISO 3166 [6]; pink violins: three countries with papers with lowest median citations yearly, blue-shaded violins: three countries with papers with highest median citations yearly).

The univariable analyses indicated the following parameters with significant association with the number of citations received per paper yearly: (a) the timespan covered in the search for records (*p* = 0.002), (b) the inclusion of specific animal species in searches (*p* = 0.037), (c) the number of cited references in papers (*p* = 0.0003) and (d) publication under open access (*p* = 0.011) (Figure 14) (Table S22). There were also differences in the number of citations received by published papers yearly among the countries with most published papers; papers from Portugal, Pakistan and China had the highest median yearly number of citations: 5.7 (IQR: 6.3), 4.7 (IQR: 1.2) and 3.5 (IQR: 3.5), respectively, whilst papers from Chile, France and India had the lowest: 0.0 (IQR: 0.7), 0.2 (IQR: 1.0) and 0.5 (IQR: 2.0) (*p* = 0.018) (Figure 15) (Table S23). In contrast, there was no difference in the number of citations received per paper yearly, in accord with the topic of assessment (*p* = 0.44 when all topics were considered, *p* = 0.35 when only the seven topics with most published papers were taken into account (Figure S7)).

**Figure 16:**
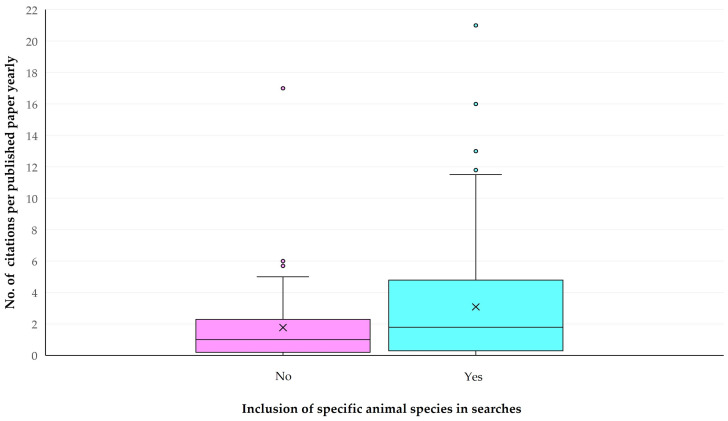
Number of citations per paper yearly, in papers on veterinary or animal studies scientometrics, in accord with the inclusion of specific animal species in the searches.

In the multivariable analysis, the following parameters emerged as significant: (a) the higher number of cited references in papers (*p* = 0.030) and (b) the inclusion of specific animal species in the searches for records (*p* = 0.045) (Figure 16, Table 2). There was also a tendency for significance for the larger number of keywords included in the published paper (*p* = 0.07).

**Figure 17:**
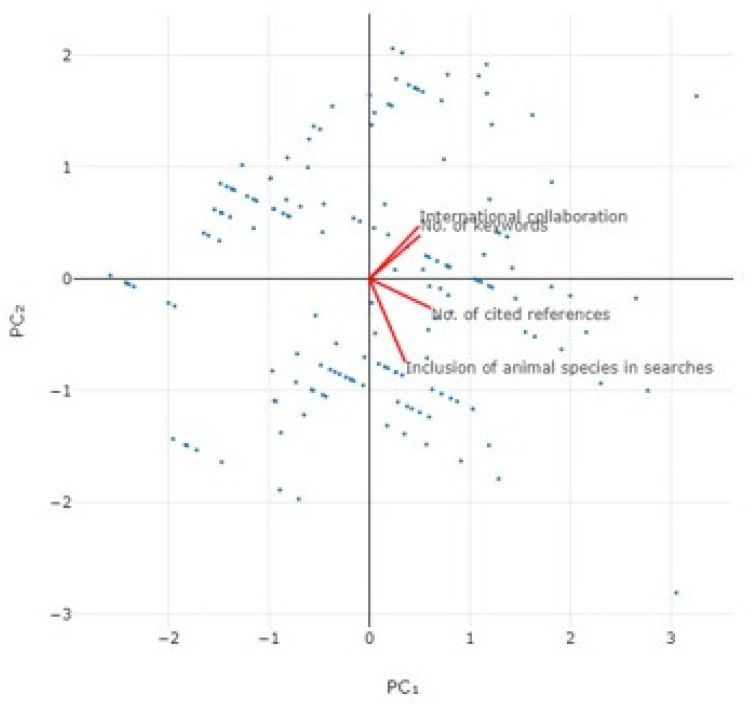
Bi-plot of results of principal component analysis for the yearly citations received by the published papers on veterinary or animal studies scientometrics.

Principal component analysis for the citations received by the published papers yearly and the four parameters included in the final multivariable assessment, revealed that the two principal components accounted for 59.9% of the variation (Figure 17 and Figure S8, Table 3).

## 4. Discussion

The idea of scientometric analyses was initiated in the 1950s and 1960s [7]. In the field of scientometrics of veterinary and animal studies, the first papers appeared in the 1980s and 1990s and were followed by sporadic publications until 2020. At that time, a massive increase in relevant publications occurred and was followed by further expansion of papers in the topic [8]. First, this increase reflects the general trend for an increased number of published papers seen across the scientific fields and the countries worldwide [9]. Moreover, we also postulate that this increase in 2020 and thereafter was triggered during the COVID-19 quarantine period, when scientists could not carry out extensive experimental or field work [10] and thus directed their interest (and energy) towards performing computational studies [11,12], as part of the general development of the information science at that period [13]. In this context, they developed and performed scientometric studies, which also answered questions pertinent to their field of research. As scientists fully appreciated the usefulness and the potential of these studies, they continued these even after all measures were lifted, leading to the continued growth of published papers.

The wide geographic expansion of the origin of published papers (the increase in author affiliations from 23 to 53 countries), the diversity of scientific organization of origin of the papers (universities, research institutes, etc.) and the proportion of papers with international collaboration lend support to our argument. Also, they point to the wide international expansion of scientometric studies.

The findings indicate that the Web of Science was the most popular database for record search, in line with the findings of Liu and He [9], who also reported that the platform was the most popular database for literature search. The Web of Science platform is a bibliometric platform unaffiliated to any publishing house, unlike other similar platforms; moreover, it applies strict quality control measures for the inclusion of journals in the database. The findings of the study have also confirmed that use of the Web of Science platform provided the largest number of records during the search. The authors of the present paper also used this database for record search in previous studies [14,15,16,17], as well as in the present one, and have found it easy to navigate and use, as well as equipped with various tools that facilitate record search and assessment.

Recently, Fassin and Rousseau [8] have reported that using the term scientometr* in an ‘ALL FIELDS’ search might yield unnecessary papers, in comparison to a ‘TOPIC’ search, due to the existence of the journal *Scientometrics*. In the present study, an ‘ALL FIELDS’ search wide approach was used. This contributed to eliminating the possibility of missing relevant records. Subsequent to using this search approach, all the records retrieved from the search were individually assessed to confirm that they were within the remit of our study. That way, superfluous papers returned from the search (‘false positives’) were eliminated during the individual assessment.

When taking into account the Web of Science categories in which journals were included, it becomes evident that there has been a shift in where papers were published. Earlier, journals related to information science were preferred for publication of relevant papers; however, in recent years, papers were published in journals concurring with the topic under assessment. This corroborates, at least to some extent, the hypothesis that scientists started included scientometric studies in the methodological approaches used in the study of their primary field of interest.

A variety of topics have been studied by means of scientometric methodologies. Most of them are directly related to veterinary science (e.g., mastitis, gastrointestinal parasitism, veterinary education); several include studies within the One Health approach (e.g., antibiotic resistance, heat stress), which is also a topic of significant veterinary involvement; and others deal with the use of animals as models for human disorders (e.g., epilepsy, tuberculosis) and also fields of veterinary work [18]. Although others have focused more on animal science (e.g., genetic improvement, precision livestock farming), it was deemed useful to include them, in order to encompass the full literature on animal-related scientometric studies.

Such, more specific, studies were found to proliferate in more recent years, in distinction to more generalized topics which had been addressed in earlier scientometric studies (country-related, specific journal-related and animal species-related studies), potentially indicating that researchers have started to include scientometric methodologies in the study of their particular fields of interest. The decrease in the number of retrieved records in correlation with the year of publication is in accord with the performance of more specific and focused scientometric studies in recent years, in more narrow topics. Consequently, the number of keywords in the published papers increased, as researchers had to include more terms of reference to describe better the topic of assessment.

In this context, the progressive increase in the number of authors in recent papers may possibly reflect the situation hypothesized above, i.e., larger groups working in various scientific topics have started scientometric analyses in their primary fields of scientific interest. Moreover, the weak connections seen between author groups confirm that the authors of the papers have focused on topics of different disciplines, which reduces the possibilities for cross-disciplinary studies on scientometrics.

The recent increase in papers published as open access papers is in line with the international trend for open science, transparency in research and availability to the public of scientific results, particularly when derived from public funds [19]. This also reflects the public requirements for expansion of open access publishing, which further promotes dissemination of scientific findings and knowledge to the academic community and the general public [20].

An increased number of citations indicates a useful study (‘reference paper’) or, alternatively, an article that has attracted attention and reference to it (in a good or bad context) [21]. Thus, high citation numbers reflect impact, but not necessarily quality [22,23]. In the same way, low citations numbers do not automatically indicate a low-quality paper, but may possibly mirror a more narrow field of study [21].

The increased number of citations found to be associated with a high number of cited references within a paper likely reflects a variety of issues dealt with in the paper and a diversity of ideas presented therein, which make it citable more easily, as the result of including more information and more hypotheses. Similar results have been reported previously by other researchers [24,25,26,27,28], as well as by our group [16]. Further, it has been suggested that such papers may become more ‘visible’ in online searches [28]. This aligns with the increased number of keywords, which reflects the diversity of ideas in the manuscript, and has been found with a tendency for association with the number of citations. Furthermore, the association of increased citations with the inclusion of specific animal species (Table S20) in the search for records aligns with a focused approach in the paper, thus making the paper visible in searches which include species-related strings.

## 5. Conclusions

The work refers to a study of ‘scientometrics of scientometrics’ focusing on the field of veterinary and animal studies. The Web of Science database was found to be the database employed most often, and also the one through which the highest number of records for analysis was obtained. The findings confirmed a clear increase in relevant papers during recent years. It is suggested that the results show a trend of including scientometrics analysis as part of the work in the primary fields of scientific interest of researchers.

There are advantages in the use of this methodological approach in veterinary and animal scientific research. Through this approach, researchers can assess large amounts of data, which have been produced independently (i.e., by other researchers) and which can provide insights in the development of a scientific field of work or a topic of research; they can also identify potential collaborators for future joint work. Thus, in all, scientometrics would be a useful tool or practice for understanding the situation in a specific research field or topic.

## Figures and Tables

**Table 1:** The most frequently assessed topics (*n* = 18) in published papers on veterinary or animal studies scientometrics and the respective number of published papers (full details in Table S14).

Topic of Assessment	Papers (*n*)
Country-related scientometrics ^1^	16
Specific journal-related scientometrics ^2^	10
Animal species-related scientometrics ^3^	10
Animal welfare	10
Genetic improvement	6
Animal ethology	5
Mastitis	5
Conservation plan	4
Antibiotic resistance	3
Gastrointestinal parasitism	3
Heat stress	3
Human–animal interactions	3
Veterinary education	3
Epilepsy	2
Infections	2
Precision livestock farming	2
Tuberculosis	2
Zoo archaeology—palaeontology	2

^1^ Full details in Table S15; ^2^ full details in Table S16; ^3^ full details in Table S17.

**Table 2:** Results of multivariable analysis for parameters with significant association with yearly citations of published papers on veterinary or animal studies scientometrics.

Parameters	Odds Risk (±se) ^1^	*p*
Cited references in papers		0.030
Per unit increase	1.021 ± 1.007	0.003
Inclusion of specific animal species in searches		0.045
No (1.0 (2.0) ^2^)	Reference	-
Yes (1.8 (4.3))	3.738 ± 1.721	0.016

^1^ se: standard error; ^2^ median (interquartile range) number of yearly citations.

**Table 3:** Eigenvalues for principal component analysis for the citations received by the published papers yearly.

Parameter	PC_1_	PC_2_	PC_3_	PC_4_
Eigenvalue	1.34	1.06	0.84	0.76
% of Variance	33.5	26.4	21.1	19.0
Cumulative variance (%)	33.5	59.9	81.0	100.0

## Data Availability

All data are available in the Web of Science platform and in the Supplementary Materials.

## Supplementary Materials

The following supporting information can be downloaded at: https://www.mdpi.com/article/10.3390/ani14213132/s1, Table S1: PRISMA flow diagram for the identification and exclusion of records from Web of Science database; Table S2: Details of a multivariable model employed for evaluation of predictors for yearly citations of papers on veterinary or animal studies scientometrics; Table S3: Details of 163 papers published until 30 June 2024 on veterinary or animal studies scientometrics and listed in the Web of Science platform; Figure S1: Proportions of published papers (a) up to 2020 and (b) from 2021 up to July 2024 among all published papers on scientometrics and among published papers on veterinary or animal studies scientometrics; Table S4: The countries of origin of published papers on veterinary or animal studies scientometrics and the respective number of published papers; Table S5: The combinations of countries of origin of published papers on veterinary or animal studies scientometrics and the respective number of published papers; Table S6: The organization of origin of published papers on veterinary or animal studies scientometrics and the respective number of published papers; Table S7: The databases used for record search in published papers on veterinary or animal studies scientometrics and the respective number of published papers, in which their use was reported; Table S8: The databases used most frequently for record search in published papers on veterinary or animal studies scientometrics and the year of publication of respective papers that reported use of database; Table S9: Frequency of published papers on veterinary or animal studies scientometrics, in accord with the country of origin of the papers and the database used for record search; Figure S2: The databases used most frequently for record search for published papers on veterinary or animal studies scientometrics and the timespan of searches in respective published papers; Table S10: The databases used most frequently for record search in published papers on veterinary or animal studies scientometrics and the number of records in respective studies; Table S11: The journals in which papers on veterinary or animal studies scientometrics were published, and the respective number of published papers; Table S12: Frequency of published papers on veterinary or animal studies scientometrics, in accord with the country of origin of the papers and the journal in which they were published; Table S13: The Web of Science categories in which journals with published papers on veterinary or animal studies scientometrics were classified, and the respective number of journals and of published papers in these journals; Table S14: The topics assessed in published papers on veterinary or animal studies scientometrics and the respective number of published papers; Table S15: The countries referred to in work reported in published papers on veterinary or animal studies scientometrics in the topic of assessment of country-related scientometrics and the respective number of published papers; Table S16: The journals referred to in work reported in published papers on veterinary or animal studies scientometrics in the topic of assessment of specific journal-related scientometrics and the respective number of published papers; Table S17: The animal species referred to in work reported in published papers on veterinary or animal studies scientometrics in the topic of assessment of specific animal species-related scientometrics and the respective number of published papers; Table S18: The topics assessed most frequently in published papers on veterinary or animal studies scientometrics and the year of publication of respective papers; Table S19: The topics assessed most frequently in published papers on veterinary or animal studies scientometrics and the number of records in respective papers; Table S20: The specific animal species referred to in papers on veterinary or animal studies scientometrics and the respective number of published papers; Figure S3: Box and whisker plot of number of records in papers on veterinary or animal studies scientometrics, in accord with the most frequently referred to specific animal species; Table S21: The keywords in papers on veterinary or animal studies scientometrics and the respective number of occurrences in the papers; Figure S4: Trendline of the progressive change of the median number of co-authors in papers on veterinary or animal studies scientometrics published yearly; Figure S5: Box and whisker plot of number of co-authors in papers on veterinary or animal studies scientometrics, among the countries with most published papers; Figure S6: Histogram of the proportion of papers published under open access among the countries with most published papers on veterinary or animal studies scientometrics; Table S22: Results of univariable analysis for associations with the number of citations received per paper on veterinary or animal studies scientometrics yearly; Table S23: The median number of yearly citations per published paper on veterinary or animal studies scientometrics from the 16 countries with most papers; Figure S7: Box and whisker plot of the yearly citations per published paper on veterinary or animal studies scientometrics in accord with the topic of study and scientometric assessment; Figure S8: Scree-plot of results of principal components analysis for the yearly citations received by the published papers on veterinary or animal studies scientometrics.
