# Supplementary material for: Scientometric Analysis: An Emerging Tool in Veterinary and Animal Scientific Research
Source: Animals (Basel). 2024 Oct 31;14(21):3132. doi: 10.3390/ani14213132 (PMC11545781; doi:10.3390/ani14213132)

Scientometric Analysis: An Emerging Tool in Veterinary and Animal Scientific Research

Georgia A. Vaitsi, Maria V. Bourganou, Daphne T. Lianou, Yiannis Kiouvrekis, Charalambia C. Michael, Dimitris A. Gougoulis and George C. Fthenakis

Table S1. PRISMA flow diagram for the identification and exclusion of records from Web of Science database.

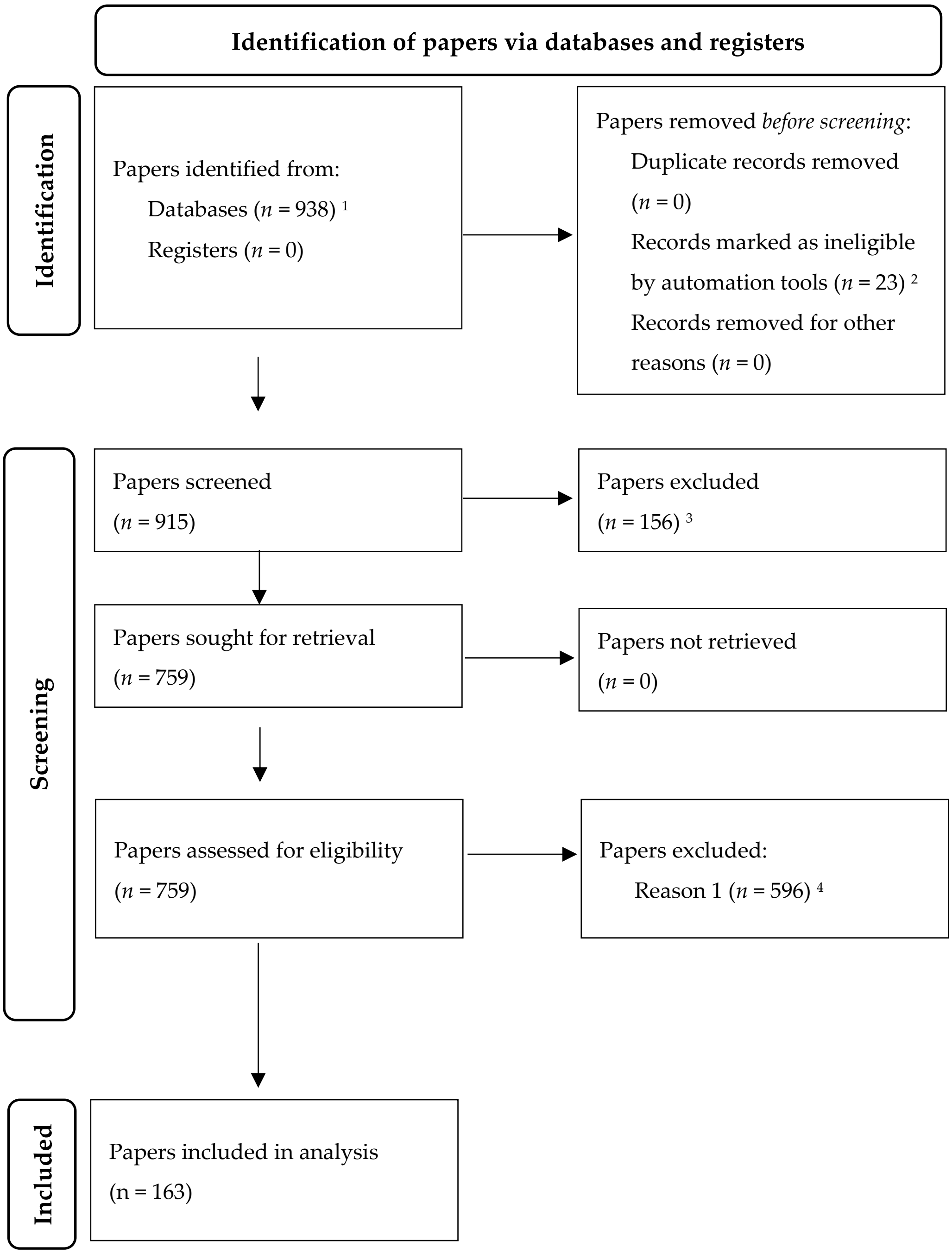

[1] Papers search performed in Web of Science database; [2] Papers excluded for not being 'Articles' or Review articles' – exclusion performed by the Web of Science platform; [3] Papers excluded as published after 30 June 2024 – exclusion performed manually; [4] Papers excluded for being outside the remit of (a) work related to veterinary or animal studies OR (b) work related to scientometric or bibliometric assessment – exclusion by means of individual assessment of papers.

Table S2. Details of a multivariable model employed for evaluation of predictors for yearly citations of papers on veterinary or animal studies scientometrics.

Outcome	Variables used in univariable analyses	Variables included into the multi-variable model	Parameters included in the final assessment
Citations received by a published paper yearly	*n* = 8	*n* = 8	(a) international collaborations in origin of paper, (b) number of cited references in published paper, (c) number of listed keywords in published paper, (d) inclusion of specific animal species in searches for records

Table S3. Details of 163 papers published until 30th June 2024 on veterinary or animal studies scientometrics and listed in the Web of Science platform.

Author(s)	Year of publication	Title of paper	Authors' keyword(s)	Bibliographical details
Alvitez-Temoche, D; del Aguila, E; Galarza-Valencia, D; Calderón, I; Espinoza-Carhuancho, F; Pacheco-Mendoza, J; Mayta-Tovalino, F	2024	Current trends and spatial-temporal dynamics of veterinary dentistry research: a scientometric study	bibliometrix; scientometrics; veterinary dentistry.	*Veterinary World* 17, 666-671
Aly, SM; ElBanna, NI; Fathi, M	2024	*Chlorella* in aquaculture: challenges, opportunities, and disease prevention for sustainable development	aquaculture; bacterial pathogens; chlorella; treatment; antibacterial	*Aquaculture International* 32, 1559-1586
Amado-Fuentes, M; Denche-Zamorano, A; Barrios-Fernandez, S; Gozalo, M	2024	Bibliometric analysis on equine-assisted interventions	equine-assisted services; horses; education; therapy; scientometrics	*Animals* 14, 1776
Bourganou, MV; Chatzopoulos, DC; Lianou, DT; Tsangaris, GT; Fthenakis, GC; Katsafadou, AI	2024	Scientometrics evaluation of published scientific papers on the use of proteomics technologies in mastitis research in ruminants	bibliometric analysis; cattle; goat; LC/MS-MS; mammary infection; mastitis; meta-research; one health; proteome; proteomics; sheep; staphylococcus; subclinical mastitis	*Pathogens* 13, 324
Bravo, JC; Ugartemendia, L; Barman, A; Rodríguez, AB; Pariente, JA; Bravo, R	2024	Bibliometric analysis on cannibalism/infanticide and maternal aggression towards pups in laboratory rodents	cannibalism; infanticide; laboratory rodents; maternal aggression	*Laboratory Animals*, 58, 240-251
de la Fuente, J; Rutaisire, J	2024	Bibliometric analysis for the identification of main limitations and future directions of vaccines for the control of ticks and tick-borne pathogens in Uganda	Africa; bibliometric; pathogen; tick; tick-borne diseases; Uganda; vaccine	*Current Research in Parasitology & Vector-Borne Diseases* 5, 100175
de Oliveira, FM; Ferraz, GAES; Andre, ALG; Santana, LS; Norton, T; Ferraz, PFP	2024	Digital and precision technologies in dairy cattle farming: a bibliometric analysis	precision livestock farming; dairy cows; technologies; bibliometric review	*Animals* 14, 1832
Geremia, E; Grande, U; Tomajoli, MTM; Petito, A; Fasciolo, G; Napolitano, G	2024	Feed production for sustainable aquaculture: a bibliometric network analysis	aquaculture; sustainability; aquafood; aquafeed; feed; food; bibliometric analysis	*Ecological Questions* 35, 1-17
Julek, E; Fagundes, VL; Zatta, JCD; Berthier, SA; Galvao, JA	2024	Honey from stingless bees in Brazil: bibliometric analysis and systematic review	food safety; food composition; quality	*Vigilancia Sanitaria Em Debate-Sociedade Ciencia & Tecnologia* 12, e02199

Krishnamoorthy, P; Lakshmi, HK; Jacob, SS; Suresh, KP; Patil, SS	2024	Dairy cattle and buffaloes harbouring gastrointestinal parasites in various zones and climatic regions established by scientometrics	buffaloes; cattle; gastrointestinal parasite; India; meta-analysis; prevalence	*Veterinary Parasitology - Regional Studies and Reports* 47,100966
McManus, C; de Albuquerque, LG; Dias, LT; Paiva, SR; Pimentel, D; Pimentel, F	2024	Livestock breeding, conservation and genomics in Brazil: who, when, where, and what	authors; bibliometric analyses; citation; coupling; livestock	*Livestock Science* 281, 105429
Schweiggart, N	2024	Mapping the role of animal welfare in tourism: examining discourses in tourism research and beyond using a bibliometric co-occurrence analysis of author keywords	bibliometrics; literature analysis; animal welfare; animal ethics; co-occurrence analysis	*Journal of Ecotourism*, 23
Sheikh, HI; Alhamadin, NII; Liew, HJ; Fadhlina, A; Wahid, MEA; Musa, N; Jalal, KCA	2024	Virulence factors of the zoonotic pathogen *Vibrio alginolyticus*: a review and bibliometric analysis	*Vibrio alginolyticus*; zoonotic bacteria; pathogenicity; cytotoxicity; VOSviewer	*Applied Biochemistry and Microbiology* 60, 514-531
Ucella, JGM; Ferreira, NS; Alves, MR; Ignacchiti, MDC; Dias Jr, AF; Resende, JA	2024	Evaluation of natural products as therapeutic alternatives for bovine mastitis and implications for future research	bibliometric analysis; antimicrobial alternatives; bioactive products; veterinary medicine	*South African Journal of Botany* 167, 310-321
Wang, HH; Han, DM; Blom, H; Dupret, V; Pan, ZH	2024	Evolving trends in vertebrate palaeontology (2013-2022): a bibliometric analysis using DeepBone and Web of Science databases	scientific trends; vertebrate palaeontology; bibliometric analysis; DeepBone database; Web of Science	*Historical Biology* 36,
Alarcón-Elbal, PM; Suárez-Balseiro, C; Holguino-Borda, J; Riggio-Olivares, G	2023	Research on medical and veterinary entomology in the insular caribbean: a bibliometric analysis	vector-borne diseases; Caribbean region; public health; study of arthropods; scientific publications; scientometrics	*International Journal of Tropical Insect Science* 43, 149-162
Altun, SK; Barlik, F; Aydemir, ME; Alkan, S	2023	A bibliometric analysis on *Dicrocoelium dendriticum*	bibliometrics; *Dicrocoelium dendriticum*; Scopus	*Iranian Journal of Parasitology* 18, 193-201
Arad, D; Peer, O; Ofri, R	2023	The 100 most-cited articles published in the veterinary ophthalmology journal between 1998 and 2022: a bibliometric study	author; corneal grafting; *h*-index; impact factor; publication; rebound tonometry	*Veterinary Ophthalmology* 26, 288-296
Azra, MN; Wong, LL; Aouissi, HA; Zekker, I; Amin, MA; Adnan, WNW; Abdullah, MF; Abd Latif, Z; Noor, MIM; Lananan, F; Pardi, F	2023	Crayfish research: a global scientometric analysis using CiteSpace	aquaculture; aquatic organism; crayfish; environmental factors; invasive	*Animals* 13, 1240

Benedetti, B; Felici, M; Costa, LN; Padalino, B	2023	A review of horse welfare literature from 1980 to 2023 with a text mining and topic analysis approach	machine learning; well-being; stress; pain; positive indicators	*Italian Journal of Animal Science* 22, 1095-1109
Bozkurt, AS	2023	Bibliometric analysis of the published studies on the kindling model between 1980 and 2023	kindling model; epilepsy; bibliometric	*European Journal of Therapeutics* 29, 188-193
Collado, EB; Martín, PT; Serena, OC	2023	Mapping human-animal interaction studies: a bibliometric analysis	animal studies; anthrozoology; bibliometric analysis; critical animal studies; human-animal interaction; human-animal studies	*Anthrozoos* 36, 137-157
Collins, SM; Hendrix, JG; Webber, QMR; Boyle, SP; Kingdon, KA; Blackmore, RJ; D'Entremont, KJN; Hogg, J; Ibáñez, JP; Kennah, JL; Lamarre, J; Mejías, M; Newediuk, L; Richards, C; Schwedak, K; Wijekulathilake, C; Turner, JW	2023	Bibliometric investigation of the integration of animal personality in conservation contexts	climate change; individual differences; invasive species; population management; reintroduction; temperament	*Conservation Biology* 37, e14021.
Cui, LH; Tang, WJ; Deng, XS; Jiang, B	2023	Farm animal welfare is a field of interest in China: a bibliometric analysis based on CiteSpace	farm animal welfare; China; research trends; research hotspots; research frontiers; bibliometric analysis; CiteSpace	*Animals* 13, 3143
De Oliveira, CDL; Ladle, RJ; Batista, VD	2023	Patterns and trends in scientific production on marine elasmobranchs: research hotspots and emerging themes for conservation	endangered; conservation; sharks; rays; scientometric	*Journal of Coastal Conservation* 27, 6
Deniz, M; De-Sousa, KT; Vieira, FMC; do Vale, MM; Dittrich, JR; Daros, RR; Hötzel, MJ	2023	A systematic review of the effects of silvopastoral system on thermal environment and dairy cows' behavioral and physiological responses	agroforestry systems; biometeorology; heat abatement; livestock farming; milking; natural shade	*International Journal of Biometeorology 67*, 409-422
Ding, WT; Li, JL; Ma, HY; Wu, YR; He, HL	2023	Science mapping of meta-analysis in agricultural science	scientometrics; science mapping; VOSviewer; CiteSpace; meta-analysis; agriculture	*Information* 14, 611
Ebensperger, LA; Aspillaga-Cid, A; Labra, A	2023	Scientific impact of Chilean-based animal behavioralists is positively associated with co-authorships from developed countries, high impact factor journals, but not with gender	behavioral ecology; international collaborations; *h*-index; gender; academic age; journal impact factor; first authorship; corresponding role	*Revista Chilena de Historia Natural* 96, 8

Frigeri, KDM; Kachinski, KD; Ghisi, ND; Deniz, M; Damasceno, FA; Barbari, M; Herbut, P; Vieira, FMC	2023	Effects of heat stress in dairy cows raised in the confined system: a scientometric review	cattle; compost barn; free-stall; systematic review; tie-stall; biometeorology; CiteSpace	*Animals* 13, 350
Gonzales-Malca, JA; Tirado-Kulieva, VA; Abanto-López, MS; Aldana-Juárez, WL; Palacios-Zapata, CM	2023	Worldwide research on the health effects of bovine milk containing A1 and A2 13-casein: unraveling the current scenario and future trends through bibliometrics and text mining	A1 milk; A2 milk; milk proteins; beta-casein; 13-Casomorphin-7; BCM-7	*Current Research in Food Science* 7, 100602
Gonzalez-Alcaide, G; Sosa, N; Shevy, L; Belinchon-Romero, I; Ramos-Rincon, JM	2023	Global research on cysticercosis and neurocysticercosis: a bibliometric analysis	cysticercosis; neurocysticercosis; *Tenia solium*; animals; human; bibliometrics	*Frontiers in Veterinary Science* 10, 1156834
Herrera-Angulo, AM; dos Santos, MVF; da Cunha, MV; de Mello, ACL; Ferreira, RLC; Diniz, WP; Mora-Luna, RE	2023	Bibliometric analysis on forage cactus production, management and use in animal nutrition: development, current status and future research	bibliometrix; bibliometric studies; nopalea; opuntia; semi-arid	*Chilean Journal of Agricultural & Animal Sciences* 39, 430-443
Huertas-López, A; Alvarez-García, G; Sánchez-Sánchez, R; Cantos-Barreda, A; Ibáñez-López, FJ; Martínez-Subiela, S; Cerón, JJ; Martfnez-Carrasco, C	2023	A systematic review and meta-analysis of the serological diagnosis of *Toxoplasma gondii* infection highlight the lack of a one health integrative research	meta-analysis; one health; serological techniques; systematic review; *Toxoplasma gondii*	*Research in Veterinary Science* 155, 137-149
Jiang, B; Tang, WJ; Cui, LH; Deng, XS	2023	Precision livestock farming research: a global scientometric review	precision livestock farming; animal welfare; bibliometrics; CiteSpace	*Animals* 13, 2096
Kandeel, M; Morsy, MA; Abd El-Lateef, HM; Marzok, M; El-Beltagi, HS; Al Khodair, KM; Albokhadaim, I; Venugopala, KN; Al-Rasheed, M; Ismail, MM	2023	A century of anticoccidial drugs: bibliometric analysis	anticoccidial; bibliometric; research; publications; analysis	*Frontiers in Veterinary Science* 10, 1157683
Kandeel, M; Morsy, MA; Abd El-Lateef, HM; Marzok, M; El-Beltagi, HS; Al Khodair, KM; Soliman, WE; Albokhadaim, I; Venugopala, KN	2023	A century of camel research: a bibliometric analysis	bibliometrics; camels; Web of Science; camel; research	*Frontiers in Veterinary Science* 10, 1157667
Krishnamoorthy, P; Lakshmi, HK; Jacob, SS; Suresh, KP; Shome, BR	2023	Scientometric analysis of gastrointestinal parasites prevalence in sheep and goats of India	gastrointestinal parasites; India; meta-analysis; prevalence; scientometric; sheep and goats	*Acta Parasitologica* 68, 496–519

Krishnan, P; Hemalatha, M; Agarwal, S; Gireesh-Babu, P; Naveena, BM; Rao, CHS	2023	Mapping the research publication trends among ICAR-animal sciences research institutes in India: Web of Science-based scientometric study	animal science; citations; ICAR; publication analysis; relative growth rate	*Indian Journal of Animal Sciences* 93, 928-935
Macartney, EL; Drobniak, SM; Nakagawa, S; Lagisz, M	2023	Evidence base for non-genetic inheritance of environmental exposures in non-human animals and plants: a map of evidence syntheses with bibliometric analysis	Environmental effects; scoping review; inter-generational inheritance; trans-generational inheritance; maternal effects; paternal effects; systematic review	*Environmental Evidence* 12, 1
Mafruchati, M; Ismail, WIW; Wardhana, AK; Fauzy, MQ	2023	Bibliometric analysis of veterinary medicine on embryo of animals in textbook in conceptualizing disease and health	conceptualization; genetic diversity and farmed animals; health and disease; literacy skills; veterinary	*Heliyon* 9, e17019
Marino, R; Petrera, F; Abeni, F	2023	Scientific productions on precision livestock farming: an overview of the evolution and current state of research based on a bibliometric analysis	precision livestock farming; smart farming; bibliometric analysis; bibliometrix R	*Animals* 13, 2280
Matos, FG; Stremel, ACA; Lipinski, LC; Cirelli, JA; dos Santos, FA	2023	A bibliometric analysis of the scientific literature on dental implants in large animal models	animal models; bibliometrics; dental implants; osseointegration; sheep; swine	*Journal of Osseointegration* 15, 256-266
Mazón-Ortiz, G; Cerda-Mejía, G; Mora-les, EG; Diéguez-Santana, K; Ruso, JM; González-Díaz, H	2023	Trends in nanoparticles for *Leishmania* treatment: a bibliometric and network analysis	nanomedicine; bibliometric analysis; network analysis; parasite diseases; leishmaniasis treatment; antiprotozoal agents	*Diseases* 11, 153
Nayak, BB; Iburahim, SA	2023	Towards a successful river dolphin conservation plan in India: a scientometric mapping of past research and current needs	dolphins; river-based	*Aquatic Ecosystem Health & Management* 26, 57-67
Nejad, JG; Vakili, R; Sobhani, E; Sangari, M; Mokhtarpour, A; Ghafari, SAH	2023	Worldwide research trends for chelates in animal science: a bibliometric analysis	chelate; intellectual structure; co-reference; keyword co-occurrence; hot topics; emerging topics	*Animals* 13, 2374
Okolie, JA; Jimoh, T; Akande, O; Okoye, PU; Ogbaga, CC; Adeleke, AA; Ikubanni, PP; Güleç, F; Amenaghawon, AN	2023	Pathways for the valorization of animal and human waste to biofuels, sustainable materials, and value-added chemicals	thermochemical conversion; biological conversion; human excreta; waste-to-energy; livestock manure	*Environments* 10, 46

Rubio, C; Gatica, F; Portila, A; Vázquez, D; Molina-García, J; Piñón, E; Rubio-Osornio, M	2023	Rats in epilepsy research: a bibliometric analysis of citations between 1969 and 2020 on experimental models in epilepsy	rat; bibliometric analysis; experimental model; seizure; epilepsy	*Cureus Journal of Medical Science* 15, e48891
Umar, E; Ikram, M; Haider, J; Nabgan, W; Haider, A; Imran, M; Nazir, G	2023	A state-of-the-art review on carbon quantum dots: prospective, advances, zebrafish biocompatibility and bioimaging in vivo and bibliometric analysis	carbon quantum dots; zebrafish; bibliometric; embryo	*Sustainable Materials and Technologies* 35, e00529
Uyanga, VA; Musa, TH; Oke, OE; Zhao, JP; Wang, XJ; Jiao, HC; Onagbesan, OM; Lin, H	2023	Global trends and research frontiers on heat stress in poultry from 2000 to 2021: a bibliometric analysis	bibliometric; chickens; growth performance; heat stress; oxidative stress; VOSviewer	*Frontiers in Physiology* 14, 1123582
Vazquez-Avendaño, JR; Cortez-Romero, C; Bravo-Vinaja, A; Ambríz-García, DA; Trejo-Córdova, A; Navarro-Maldonado, MD	2023	Reproduction of sheep through nuclear transfer of somatic cells: a bibliometric approach	SCNT; nuclear transfer; reproductive biotechnology; bibliometric analysis; sheep; VOSviewer	*Animals* 13, 1839
Vieira, RA; McManus, C	2023	Bibliographic mapping of animal genetic resources and climate change in farm animals	animal adaptation; animal mapping; animal production; heat stress; VOSviewer	*Tropical Animal Health and Production* 55, 259
Yardibi, F; Chen, CM; Firat, MZ; Karacaören, B; Süzen, E	2023	The trend of breeding value research in animal science: bibliometric analysis	--- [1]	*Archives Animal Breeding* 66, 163-181
Yu, ZY; Xie, L; Shuai, PQ; Zhang, J; An, W; Yang, M; Zheng, J; Lin, H	2023	New perspective on African swine fever: a bibliometrics study and visualization analysis	African swine fever; bibliometrics; citation; Web of Science; visualized analysis	*Frontiers in Veterinary Science* 10, 1085473
Zaib, G; Hu, XM; Cui, HM	2023	Global maps of avian leukosis viruses: research trends and themes based on networking	avian leukosis viruses; bibliometric analysis; bibliometric-ALV; research proliferation; VOSviewer; biblioshiny; network analysis	*Veterinary Sciences* 10, 16
Banchi, P; Rota, A; Bertero, A; Domain, G; Hassan, HA; Lannoo, J; Van Soom, A	2022	Trends in small animal reproduction: a bibliometric analysis of the literature	small animal; reproduction; canine; feline; bibliometric	*Animals* 12, 336
Cao, R; Li, J; Koyabu, D	2022	A bibliometric analysis of research trends in bat echolocation studies between 1970 and 2021	bat echolocation; Bibliometric analysis; VOSviewer; evolution	Ecological Informatics 69, 101654

Chen, J; Liu, J; Liu, X; Zeng, CD; Chen, Z; Li, SF; Zhang, Q	2022	Animal model contributes to the development of intracranial aneurysm: a bibliometric analysis	intracranial aneurysms; animal model; bibliometrics; VOSviewer; CiteSpace; keyword co-occurrence analysis	*Frontiers in Veterinary Science* 9, 1027453
Chen, R; Liu, Z; Wang, JZ; Jin, WG; Abdu, HI; Pei, JJ; Wang, Q; Abd El-Aty, AM	2022	A review of the nutritional value and biological activities of sturgeon processed byproducts	sturgeon; nutrients; byproducts; processing; biological activities	*Frontiers in Nutrition* 9, 1024309
Chen, ZF; Hsu, YHE; Lee, JJ; Chou, CH	2022	Bibliometric analysis of veterinary communication education research over the last two decades: rare yet essential	veterinary education; communication; veterinarians; clients; bibliometrics; VOSviewer; Web of Science Core Collection database	*Veterinary Sciences* 9, 256
Costa, RD; Medeiros, AN; do Amaral, VS; Navoni, JA	2022	Bibliometric analysis of the ecotoxicological effects of ivermectin	antiparasitic; pharmaceutical pollution; drug pollution; environmental health; COVID-19	*Revista de Salud Ambiental* 22, 208-216
Domingues, K; Franco, NH; Rodrigues, I; Stilwell, G; Magalhaes-Sant'Ana, M	2022	Bibliometric trend analysis of non-conventional (alternative) therapies in veterinary research	literature search; text mining; evidence-based medicine; complementary therapies; alternative therapies; acupuncture; phytotherapy; homeopathy; essential oils; veterinary medicine	*Veterinary Quarterly* 42, 192-198
Domínguez-Odio, A; Pérez, O; Batista-Duharte, A; Cala-Delgado, DL	2022	Technology surveillance in veterinary vaccine adjuvants (2015-2022): university-industry interaction	adjuvant; immunoprophylaxis; technological surveillance; veterinary vaccine	*Journal of Pharmacy & Pharmacognosy Research* 10, 875-887
Emelyanova, A; Savolainen, A; Oksanen, A; Nieminen, P; Loginova, O; Abass, K; Rautio, A	2022	Research on selected wildlife infections in the circumpolar arctic-a bibliometric review	Arctic; bibliometric review; infectious disease; one health; wildlife health; zoonosis	*International Journal of Environmental Research and Public Health* 19, 11260
Farooq, M; Khan, AU; El-Adawy, H; Mertens-Scholz, K; Khan, I; Neubauer, H; Ho, YS	2022	Research trends and hotspots of Q Fever research: a bibliometric analysis 1990-2019	---	*Biomed Research International* 2022, 9324471
García-Alcalde, M; Minaya, D; Ortega, J; Alvariño, L; Iannacone, J	2022	Bibliometric analysis of theses of parasites in terrestrial fauna in the universities of Peru	*mammalia*; observational research; scientific article; veterinary medicine; virtual repository	*Revista de Investigaciones Veterinarias del Peru* 33, e22587
Gómez-Quispe, O; Benites, RM; Contreras, W; Ibañez, V	2022	Theses developed, time of completion and publication as scientific papers in a university faculty of veterinary medicine and zootechnics of Peru	advisor; bibliometrics; publication; student; undergraduate	*Revista de Investigaciones Veterinarias del Peru* 33, e23345

Jiménez-Montenegro, L; Alfonso, L; Mendizabal, JA; Urrutia, O	2022	Worldwide research trends on milk containing only A2 β-Casein: a bibliometric study	milk proteins; beta-casein; A2 milk; BCM-7; bovine	*Animals* 12, 1909
Kophamel, S; Illing, B; Ariel, E; Difalco, M; Skerratt, LF; Hamann, M; Ward, LC; Méndez, D; Munns, SL	2022	Importance of health assessments for conservation in noncaptive wildlife	biodiversity hotspots; conceptual framework; diagnostic techniques; red lists; sample size; systematic review; vertebrates; listas rojas; marco conceptual; hotspots de biodiversidad; revisió n sistemá tica; tamañ o muestral; té cnicas diagnó sticas; vertebrados	*Conservation Biology* 36
Krishnamoorthy, P; Lakshmi, HK; Siju, SJ; Suresh, KP; Shome, BR	2022	A scientometric study on prevalence of gastrointestinal parasites in pigs (Sus scrofa) of India br	gastrointestinal parasites; India; Meta -analysis; pigs; prevalence; systematic review	*Indian Journal of Animal Sciences* 92, 1264-1273
Leal, W; Ternova, L; Parasnis, SA; Kovaleva, M; Nagy, GJ	2022	Climate change and zoonoses: a review of concepts, definitions, and bibliometrics	zoonosis; climate change; health hazards; humans; animals; bibliometric analysis	*International Journal of Environ-mental Research and Public Health* 19, 893
Lianou, DT; Fthenakis, GC	2022	Scientometrics study of research output on sheep and goats from Greece	goat; mastitis; meta-research; milk production; research analysis; research assessment; research mapping; sheep; small ruminants; veterinary	*Animals* 12, 2666
Miao, LY; Li, H; Ding, W; Lu, SN; Pan, SN; Guo, XK; Zhou, XN; Wang, DQ	2022	Research priorities on one health: a bibliometric analysis	one health; bibliometric analysis; research priority; research status; research hotspots	*Frontiers in Public Health* 10, 889854
Munro, BA; Bergen, P; Pang, DSJ	2022	Randomization, blinding, data handling and sample size estimation in papers published in veterinary anaesthesia and analgesia in 2009 and 2019	arrive; bias; bibliometrics; consort; reporting guidelines	*Veterinary Anaesthesia and Analgesia* 49, 18-25
Nandutu, I; Atemkeng, M; Okouma, P	2022	Intelligent systems using sensors and/or machine learning to mitigate wildlife-vehicle collisions: a review, challenges, and new perspectives	wildlife-vehicle collisions; intelligent systems; sensor; machine learning; human-wildlife; human behavior; animal behavior; machine learning datasets; animal detection systems	*Sensors* 22, 2478
Padilla-Navarro, P; Vallejos-Romero, A	2022	Scientific articles in Chilean agricultural sciences 1989 - 2016: evolution, disciplines and impact in terms of leading in their field	bibliometrics; Chile; agricultural science; scientific collaboration; internationalization; scientific production	*Agricultura Sociedad y Desarrollo* 19, 168-183

Riaño, HB; Escobar, JW; Linfati, R; Ortiz-Araya, V	2022	Disciplinary categorization of the cattle supply chain-a review and bibliometric analysis	supply chain; cattle; sustainability; categorization	*Sustainability* 14, 14275
Silva, BBI; Urzo, MLR; Encabo, JR; Simbulan, AM; Lunaria, AJD; Sedano, SA; Hsu, KC; Chen, CC; Tyan, YC; Chuang, KP	2022	Pigeon circovirus over three decades of research: bibliometrics, scoping review, and perspectives	bibliometrics; circovirus; pigeon circovirus; young pigeon disease syndrome	Viruses-Basel 14, 1498
Silva, GGBS; Ferraz, PFP; Damasceno, FA; Zotti, MLAN; Barbari, M	2022	Compost barns: a bibliometric analysis	dairy cattle; dairy cow; compost-bedded pack barn	*Animals* 12, 2492
Sofyantoro, F; Yudha, DS; Lischer, K; Nuringtyas, TR; Putri, WA; Kusuma, WA; Purwestri, YA; Swasono, RT	2022	bibliometric analysis of literature in snake venom-related research worldwide (1933-2022)	snake venom; bibliometry; VOSviewer	Animals 12, 2058
Su, KW; Zhang, H; Lin, L; Hou, YL; Wen, YL	2022	Bibliometric analysis of human-wildlife conflict: from conflict to coexistence	human-wildlife conflict; bibliometric analysis; VOSviewer software; bibliometrix	*Ecological Informatics* 68, 101531
Suresh, KP; Bhavya, AP; Shivamallu, C; Achar, RR; Silina, E; Stupin, V; Kollur, SP; Shome, BR; Patil, SS	2022	Seroprevalence of sheeppox and goatpox virus in Asia and African continent: a systematic review and meta-analysis (Scientometrics)	Asia; Africa; capripoxvirus; meta-analysis; seroprevalence; subgroup analysis	*Veterinary World* 15, 455-464
Vaziri, E; Maghsoudi, A; Feizabadi, M; Faraji-Arough, H; Rokouei, M	2022	Scientometric evaluation of 100-year history of poultry science (1921-2020)	key words; content analyses; subject areas; collaboration; research fronts; scientometrics	*Poultry Science* 101, 102134
Wu, R; Yakhkeshi, S; Zhang, XY	2022	Scientometric analysis and perspective of IgY technology study	scientometric analysis; egg yolk immunoglobulin; IgY technology; poultry	*Poultry Science* 101, 101713
Zebakh, S; Rhouma, A; Arvanitis, R; Sadiki, M	2022	Mapping the agricultural research systems in the Maghreb (Algeria, Morocco and Tunisia)	agricultural research; bibliometric; Algeria; Morocco; Tunisia; scientific production; research policy	*Science Technology and Society* 27, 429-455
Ahmad, T; Haroon; Khan, M; Murad, MA; Baig, M; Murtaza, BN; Khan, MM; Harapan, H; Hui, J	2021	Research trends in rabies vaccine in the last three decades: a bibliometric analysis of global perspective	rabies; vaccine; bibliometric analysis	*Human Vaccines & Immunotherapeutics* 17, 3169-3177

Ahmad, T; Imran, M; Ahmad, K; Khan, M; Baig, M; Al-Rifai, RH; Al-Omari, B	2021	A bibliometric analysis and global trends in fascioliasis research: a neglected tropical disease	fascioliasis; bibliometric analysis; research trend; web of science core collection; VOSviewer software	*Animals* 11, 3385
Aria, M; Alterisio, A; Scandurra, A; Pinelli, C; D'Aniello, B	2021	The scholar's best friend: research trends in dog cognitive and behavioral studies	dog; bibliometrix; behavioral science; science mapping; cognition; behavior	*Animal Cognition* 24, 541-553
Colombino, E; Prieto-Botella, D; Capucchio, MT	2021	Gut health in veterinary medicine: a bibliometric analysis of the literature	gut health; veterinary medicine; bibliometric analysis	*Animals* 11, 1997
Della Corte, V; Del Gaudio, G; Sepe, F; Nevola, G	2021	Kosher meat and production issues: a bibliometric analysis	kosher; meat; slaughter process; animal welfare; sustainability	*Trends in Food Science & Technology* 116, 749-754
Di Cosmo, A; Pinelli, C; Scandurra, A; Aria, M; D'Aniello, B	2021	Research trends in octopus biological studies	cephalopods; model species; bibliometrix; bibliometric analysis; science mapping	*Animals* 11, 1808
Fakhar, M; Keighobadi, M; Hezarjaribi, HZ; Montazeri, M; Banimostafavi, ES; Sayyadi, S; Hamadani, MMG; Sharifpour, A; Tabaripour, R; Asadi, S; Soosaraei, M; Khasseh, AA	2021	Two decades of echinococcosis/hydatidosis research: bibliometric analysis based on the web of science core collection databases (2000-2019)	bibliometric; scientific collaboration; echinococcosis; hydatidosis; *Echinococcus granulosus sensu lato*; *Echinococcus multilocularis*; Web of Science	*Food and Waterborne Parasitology* 25, e00137
Freire, R; Massaro, M; McDonald, S; Trathan, P; Nicol, CJ	2021	A citizen science trial to assess perception of wild penguin welfare	animal welfare; spheniscidae; anthropogenic impact; five domains model; welfare assessment	*Frontiers in Veterinary Science* 8, 698685
Frias-De-Diego, A; Jara, M; Pecoraro, BM; Crisci, E	2021	Whole genome or single genes? a phylodynamic and bibliometric analysis of PRRSV	bibliometrics; phylodynamics; pig; PRRSV; ORF5; whole genome	*Frontiers in Veterinary Science* 8, 658512
Garg, KC; Kumar, S; Bansal, S	2021	Bibliometrics of Indian veterinary science research output during 2001-2020	bibliometrics; scientometrics; veterinary science; citation analysis; India	*Annals of Library and Information Studies* 68, 411-421
Guerrero-Casado, J; Monge-Nájera, J	2021	The geographic bias of mammal studies: a comparison of a half a century of research on palearctic and neotropical mammals	citation; geographical bias; mammalia; research impact; scientometrics; wildlife conservation	*Revista de Biologia Tropical* 69, 391-402
Gupta, BM; Surulinathi, M; Ahmed, KKM	2021	Animal models in Covid-19 research: a scientometric assessment of Indian publications during 2020-21	Covid-19; animal models; global publications; Indian publications; bibliometrics; scientometrics1	*Journal of Young Pharmacists* 13, S52-S58

Krishnamoorthy, P; Suresh, KP; Jayamma, KS; Shome, BR; Patil, SS; Amachawadi, RG	2021	An understanding of the global status of major bacterial pathogens of milk concerning bovine mastitis: a systematic review and Meta-Analysis (scientometrics)	*Staphylococcus* species; *Streptococcus* species; *Escherichia coli*; prevalence; world; systematic review; meta-analysis	*Pathogens* 10, 545
Li, ZQ; Hu, MH; Song, HT; Lin, DH; Wang, YJ	2021	Toxic effects of nano-TiO2 in bivalves-a synthesis of meta-analysis and bibliometric analysis	Meta-analysis; titanium dioxide nanoparticles; toxic effect; bivalves; bibliometric analysis	*Journal of Environmental Sciences* 104, 188-203
Martin, JM; Bertram, MG; Blanchfield, PJ; Brand, JA; Brodin, T; Brooks, BW; Cerveny, D; Lagisz, M; Ligocki, IY; Michelangeli, M; Nakagawa, S; Orford, JT; Sundin, J; Tan, H; Wong, BBM; McCallum, ES	2021	Evidence of the impacts of pharmaceuticals on aquatic animal behaviour: a systematic map protocol	medicine; neurotoxicology; psychoactive; ecotoxicology; evidence synthesis; fitness	*Environmental Evidence* 10, 26
Nolen, DS; Kathuria, S; Peacock, E	2021	Quantifying interdisciplinarity: subject librarians as research collaborators	applied zooarchaeology; citation analysis; bibliometrics; scholarly communication; interdisciplinary	*Journal of Academic Librarianship* 47, 102419
Olias-Molero, AI; Fontán-Matilla, E; Cuquerella, M; Alunda, JM	2021	Scientometric analysis of chemotherapy of canine leishmaniasis (2000-2020)	allopurinol; amphotericin; antimonials; canine leishmaniasis; chemotherapy; *Leishmania* spp; *Leishmania infantum*; miltefosine; Sb-V	*Parasites & Vectors* 14, 36
Reis, AC; Ramos, B; Pereira, AC; Cunha, MV	2021	Global trends of epidemiological research in livestock tuberculosis for the last four decades	animal tuberculosis; bibliometric review; epidemiology; livestock; livestock production system; one health; research trends	*Transboundary and Emerging Diseases* 68, 333-346
Reis, AC; Ramos, B; Pereira, AC; Cunha, MV	2021	The hard numbers of tuberculosis epidemiology in wildlife: a meta-regression and systematic review	animal tuberculosis; bibliometric review; meta-analysis; one Health; research trends; wildlife	*Transboundary and Emerging Diseases* 68, 3257-3276
Schoenfeld-Tacher, RM; Alpi, KM	2021	A 45-year retrospective content analysis of JVME articles	educational research methods; veterinary education; educational scholarship; bibliometric analysis	*Journal of Veterinary Medical Education* 48, 729-746
Sweileh, WM	2021	Global research activity on antimicrobial resistance in food-producing animals	food-producing animals; antimicrobial resistance; bibliometric analysis; Scopus	*Archives of Public Health* 79, 49

Wirth, W; Lesbarrères, D; Ariel, E	2021	Ten years of Ranavirus research (2010-2019): an analysis of global research trends	Ranavirus; scientometrics; bibliometrics; collaboration	*Facets* 6, 44-57
Yardibi, F; Firat, MZ; Teke, EÇ	2021	Trend topics in animal science: a bibliometric analysis using CiteSpace	bibliometric analysis; citespace; animal science literature; social network analysis; citation analysis	*Turkish Journal of Veterinary & Animal Sciences* 45, 833-840
Yatcilla, JK	2021	A panorama of human-animal interactions research: bibliometric analysis of HAI articles 1982-2018	bibliometric analysis; bibliometrics; human–animal interaction; text analytics	*Anthrozoos* 34, 161-173
Alpi, KM; Stafford, E; Swift, EM; Danehower, S; Paxson, HI; Davidson, G	2020	Characterization of veterinary pharmacy and pharmacology literature and its availability to pharmacy education	veterinary pharmacy; drug monographs; abstracts and indexing; bibliometrics; pharmacy libraries	*American Journal of Pharmaceutical Education* 84, 7314
Andreo-Martínez, P; Oliva, J; Giménez-Castillo, JJ; Motas, M; Quesada-Medina, J; Cámara, MA	2020	Science production of pesticide residues in honey research: a descriptive bibliometric study	bibliometric review; honey; honey-bees; pesticide; residues	*Environmental Toxicology and Pharmacology* 79, 103413
da Frota, AVB; Vitorino, BD; Nunes, JRD; da Silva, CJ	2020	Main trends and gaps in studies for bird conservation in the pantanal wetland	---	*Neotropical Biology and Conservation* 15, 427-445
Frias-De-Diego, A; Posey, R; Pecoraro, BM; Carnevale, RF; Beaty, A; Crisci, E	2020	A century of swine influenza: is it really just about the pigs?	bibliometrics; swine influenza virus; influenza variants; GenBank; global health	*Veterinary Sciences* 7, 189
González, M; Salgado-Arroyo, L	2020	Bibliometric analysis of the scientific articles published in veterinary medicine and zootechnics in Colombia 2010-2019	information analysis; scientific press; veterinary; popularising science	*Revista MVZ Cordoba* 25, e2114
Jara, M; Frias-De-Diego, A; Machado, G	2020	Phylogeography of equine infectious anemia virus	equine anemia; infectious diseases; molecular epidemiology; phylodynamics; viral evolution	*Frontiers in Ecology and Evolution* 8, 127
Lianou, DT; Fthenakis, GC	2020	Scientometrics approach to research in ovine mastitis from 1970 to 2019 (with a complete list of relevant literature references)	ewe; intramammary infection; mastitis; meta-research; ovine; scientometrics; sheep; subclinical mastitis	*Pathogens* 9, 585
Maghsoudi, A; Vaziri, E; Feizabadi, M; Mehri, M	2020	Fifty years of sheep red blood cells to monitor humoral immunity in poultry: a scientometric evaluation	chicken; humoral immunity; immune responses; SRBC; broilers; medicinal plants	*Poultry Science* 99, 4758-4768
Manuelian, CL; Penasa, M; da Costa, L; Burbi, S; Righi, F; De Marchi, M	2020	Organic livestock production: a bibliometric review	bibliometrix R; cattle; organic; poultry; sheep	*Animals* 10, 618

Pastrana, CI; González, FJN; Ciani, E; Capote, CJB; Bermejo, JVD	2020	Effect of research impact on emerging camel husbandry, welfare and social-related awareness	animal welfare; bibliometrics; camels; emerging industry; international research; science-society dialogue; law enforcement	*Animals* 10, 780
Phillips, CJC; Molento, CFM	2020	Animal welfare centres: are they useful for the improvement of animal welfare?	animal wellbeing; animal welfare; animal welfare science; centres of animal welfare	*Animals* 10, 877
Pyott, BE; Schulte-Hostedde, AI	2020	Peer-reviewed scientific contributions from Canadian zoos and aquariums	zoos; conservation; publications; bibliometrics	*Facets* 5, 381-392
Reichel, MP; Wahl, LC; Ellis, JT	2020	Research into *neospora caninum*-what have we learnt in the last thirty years?	*neospora caninum*; dogs; cattle; abortions; diagnosis; pathogenesis; review	*Pathogens* 9, 505
Resende, PS; Viana, AB; Young, RJ; de Azevedo, CS	2020	A global review of animal translocation programs	bibliometric analysis; conservation; hard release; knowledge gap; soft release	*Animal Biodiversity and Conservation* 43, 221-232
Silva, DC; Queiroz, PJB; Borges, PAC; Pedroso, ACBD; Arnhold, E; da Cruz, AS; da Cruz, AD; Silva, LAF	2020	Half a century of research on cattle foot and claw diseases: a scientometric analysis	bovine; digital dermatitis; hoof disorder; laminitis; sole ulcer	*Semina - Ciencias Agrarias* 41, 223-235
Sinclair, M; Zhang, Y; Descovich, K; Phillips, CJC	2020	Farm animal welfare science in China-a bibliometric review of Chinese literature	animal welfare; China; science; bibliometric review; livestock production; Asia; pigs; poultry	*Animals* 10, 540
Torres, RT; Carvalho, J; Cunha, MV; Serrano, E; Palmeira, JD; Fonseca, C	2020	Temporal and geographical research trends of antimicrobial resistance in wildlife - a bibliometric analysis	AMR; bibliometric analysis; wildlife; one health; *E. coli*; Genes	*One Health* 11, 100198
Trigueiro, NSD; Canedo, A; Braga, DLD; Luchiari, AC; Rocha, TL	2020	Zebrafish as an emerging model system in the global south: two decades of research in Brazil	*Danio rerio*; animal model; research networks; bibliometric analysis	*Zebrafish* 17, 412-425
Bauer, H; Gebresenbet, F; Kiki, M; Simpson, L; Sillero-Zubiri, C	2019	Race and gender bias in the research community on African lions	panthera leo; black; African; women; author; diversity; representation; inclusion	*Frontiers in Ecology and Evolution* 7, 24
Freire, R; Nicol, CJ	2019	A bibliometric analysis of past and emergent trends in animal welfare science	animal welfare; animal well-being; bibliometrics; citation analysis; publications; scientometrics	*Animal Welfare* 28, 465-485
Gautret, M; Messori, S; Jestin, A; Bagni, M; Boissy, A	2017	Development of a semi-automatic bibliometric system for publications on animal health and welfare: a methodological study	animal health and welfare; bibliometrics; database accuracy; research coordination	*Scientometrics* 113, 803-823

Pang, DSJ	2019	Misconceptions surrounding the relationship between journal impact factor and citation distribution in veterinary medicine	bibliometrics; citation; journal impact factor; metrics	*Veterinary Anaesthesia and Analgesia* 46, 163-172
Singh, N; Datta, SN; Handa, TS	2019	Research dynamics in Indian fisheries and aquaculture: a scientometric analysis	Aquaculture; fisheries; chronological growth; international collaboration; network visualization; scientometric study	*Current Science* 117, 382-389
Staubli, N; Schmidt, JC; Rinne, CA; Signer-Buset, SL; Rodriguez, FR; Walter, C	2019	Animal experiments in periodontal and peri-implant research: are there any changes?	animal experiments; bibliometrics; periodontal and peri-implant research; journal of periodon-tology; journal of clinical periodontology	*Dentistry Journal* 7, 46
Zheng, CL; Cotner, JB; Sato, C; Li, G; Xu, YY	2018	Global development of the studies focused on antibiotics in aquatic systems from 1945 to 2017	antibiotics; aquatic systems; cross-disciplines; international collaborations; research trends	*Environmental Science and Pollution Research* 25, 22023-22034
Friese, C; Nuyts, N	2017	Posthumanist critique and human health: how nonhumans (could) figure in public health research	posthumanism; nonhumans; animals; one-health; bibliometric analysis	*Critical Public Health* 27, 303-313
Krauskopf, E; Garcia, F; Funk, R	2017	Bibliometric analysis of multi-language veterinary journals	bibliometric; impact factor; multi-language; scientific journal; veterinary science	*Transinformacao* 29, 343-352
Yoon, SJ; Yoon, DY; Cho, YK; Baek, S; Lim, KJ; Seo, YL; Yun, EJ	2017	Characteristics and quality of published animal research in the field of radiology	bibliometrics; publication; radiologists; animal research; quality assessment	*Acta Radiologica* 58, 685-691
Díaz, I; Cortey, M; Olvera, A; Segalés, J	2016	Use of *h*-index and other bibliometric indicators to evaluate research productivity outcome on swine diseases	---	*Plos One* 11, e0149690
Ducrot, C; Gautret, M; Pineau, T; Jestin, A	2016	Research carried out in France on infectious diseases affecting livestock animals: temporal trend and comparison with international research	---	*INRA Productions Animales* 29, 75-85
Ducrot, C; Gautret, M; Pineau, T; Jestin, A	2016	Scientific literature on infectious diseases affecting livestock animals, longitudinal worldwide bibliometric analysis	---	*Veterinary Research* 47, 42
Rodriguez-Ledesma, A; Cobo, MJ; Lopez-Pujalte, C; Herrera-Viedma, E	2015	An overview of animal science research 1945-2011 through science mapping analysis	animal science; bibliometric network; coword analysis; *h*-index; knowledge visualisation; science mapping analysis	*Journal of Animal Breeding And Genetics* 132, 475-497

Sweileh, WM; Al-Jabi, SW; Sawalha, AF; AbuTaha, AS; Zyoud, SH	2016	Bibliometric analysis of publications on *Campylobacter:* (2000-2015)	*campylobacter*; bibliometrics	*Journal of Health Population and Nutrition* 35, 39
Gupta, BM; Ahmed, KKM; Gupta, R; Tiwari, R	2015	World camel research: a scientometric assessment, 2003-2012	camel research; scientometrics; global; publication output	*Scientometrics* 102, 957-975
Nascimento, FD; Rodrigues, FM	2015	Growth trend of scientific literature on genetic improvement through the database Scopus	scientometrics; genetic improvement; Scopus; breeding	*Scientometrics* 105, 805-816
Page, JR; Moberly, HK; Youngen, GK; Hamel, BJ	2014	Exploring the veterinary literature: a bibliometric methodology for identifying interdisciplinary and collaborative publications	---	*College & Research Libraries* 75, 664-683
Christopher, MM; Marusic, A	2013	Geographic trends in research output and citations in veterinary medicine: insight into global research capacity, species specialization, and interdisciplinary relationships	agriculture; bibliometrics; economics; education; journal; medicine; research publication	*BMC Veterinary Research* 9, 115
Spilki, FR	2013	Profile of the conselho nacional de desenvolvimento cientifico e tecnologico (CNPq) productivity fellows in the area of veterinary medicine	research productivity; veterinary medicine; scientometrics	*Pesquisa Veterinaria Brasileira* 33, 205-213
Chen, L; Dou, YX; Cai, XP	2012	A bibliometric analysis of peste des petits ruminants	PPR; bibliometrics; publications; financial support; China	*Journal of Animal and Veterinary Advances* 11, 4389-4393
Malesios, C; Abas, Z	2012	Examination of the impact of animal and dairy science journals based on traditional and newly developed bibliometric indices	animal and dairy science journals; journal evaluation journal *h*-index; journal impact factor	*Journal of Animal Science* 90, 5170-5181
Hessels, LK; Grin, J; Smits, REHM	2011	The effects of a changing institutional environment on academic research practices: three cases from agricultural science	---	*Science and Public Policy* 38, 555-568
Spivak, ED	2010	The crab neohelice (=*chasmagnathus*) granulata: an emergent animal model from emergent countries	emergent animal model; crab; Argentina; Brasil	*Helgoland Marine Research* 64, 149-154
Carreño, LM; Poutou-Piñales, RA; Mattar, S; González, M	2009	Bibliometric activity indicators for the journal *MVZ Cordoba* 1994-2008	Bibliometric analysis; price index; insularity index; productivity index; colaboration index; research; journal; Colombia	*Revista MVZ Cordoba* 14, 1531-1543
Goulart, VD; Azevedo, PG; van de Schepop, JA; Teixeira, CP; Barçante, L; Azevedo, CS; Young, RJ	2009	GAPs in the study of zoo and wild animal welfare	animal well-being; GAP analysis; wild animal; zoo animal	*Zoo Biology* 28, 561-573

Anderson, US; Kelling, AS; Maple, TL	2008	Twenty-five years of zoo biology: a publication analysis	authorship; bibliometrics; citations; research; scientific communication	*Zoo Biology* 27, 444-457
Pulina, G; Francesconi, AHD	2007	Some bibliometric indexes for members of the scientific association of animal production (ASPA)	bibliometry; scientometry; research; evaluation; ASPA	*Italian Journal of Animal Science* 6, 83-103
Crawley-Low, J	2006	Bibliometric analysis of the american journal of veterinary research to produce a list of core veterinary medicine journals	---	*Journal of the Medical Library Association* 94, 430-434
Garg, KC; Kumar, S; Lal, K	2006	Scientometric profile of indian agricultural research as seen through science citation index expanded	---	*Scientometrics* 68, 151-166
Clement, F; Bassecoulard, E	2004	A bibliographic study about equine research.	---	*INRA Productions Animales* 17, 69-76
Pelzer, NL; Wiese, WH	2003	Bibliometric study of grey literature in core veterinary medical journals	---	*Journal of the Medical Library Association* 91, 434-441
Rivas, AL; Wilson, DJ; Gonzalez, RN; Mohammed, HO; Quimby, FW; Lein, DH; Milligan, RA; Colle, RD; Deshler, JD; Trochim, WMK	1997	An interdisciplinary and systems-based evaluation of academic programs: bovine mastitis-related veterinary research, education and outreach	---	*Scientometrics* 40, 195-213
Ungar, K	1997	A bibliometric evaluation of the performance of ATLA	---	*ATLA-Alternatives to Laboratory Animals* 25, 67-69
Nederhof, AJ; Meijer, RF; Moed, HF; Vanraan, AFJ	1993	Research performance indicators for university departments - a study of an agricultural university	---	*Scientometrics* 27, 157-178
Pouris, A	1989	A scientometric assessment of agricultural-research in South Africa	---	*Scientometrics* 17, 401-413
King, J	1988	The use of bibliometric techniques for institutional research evaluation - a study of avian virology research	----	*Scientometrics* 14, 295-313

[1] keywords not included.

Figure S1. Proportions of published papers (a) up to 2020 (yellow colour) and (b) from 2021 up to July 2024 (blue colour) among all published papers on scientometrics (left pie-chart) and among published papers on veterinary or animal studies scientometrics (right pie-chart) (up to 2020: up to the end of the year; 2024: up to the end of June 2024).

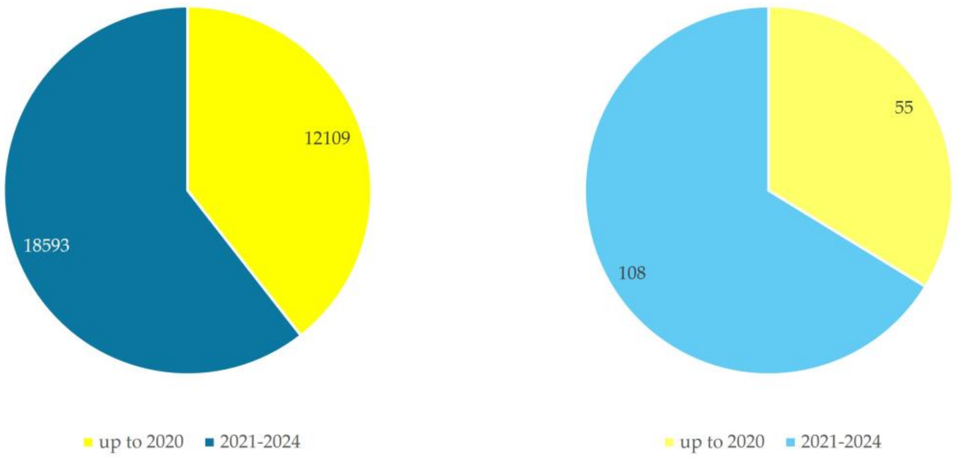

Table S4. The countries (n = 58) of origin of published papers on veterinary or animal studies scientometrics and the respective number of published papers.

Country	No. of published papers (n = 163)
United States of America	24
Brazil	22
China	20
Spain	18
India	13
Italy	13
United Kingdom	11
Australia	9
Canada	8
Chile	6
Egypt	5
France	5
Iran	5
Pakistan	5
Portugal	5
Turkiye	5
Colombia	4
Germany	4
Greece	4
Indonesia	4
Peru	4
Saudi Arabia	4
South Korea	4
Malaysia	3
Mexico	3
Poland	3
South Africa	3
Chinese Taiwan	3
Belgium	2
Ecuador	2
Nigeria	2
'Palestine'	2
Russia	2
Sweden	2
The Netherlands	2
Algeria	1
Argentina	1
Benin	1
Brunei	1
Costa-Rica	1

Croatia	1
Cuba	1
Czech Republic	1
Dominican Republic	1
Estonia	1
Finland	1
Israel	1
Japan	1
Morocco	1
Norway	1
Philippines	1
Sudan	1
Switzerland	1
Tunisia	1
Uganda	1
United Arab Emirates	1
Uruguay	1
Venezuela	1

Table S5. The combinations of countries (n = 52) of origin of published papers on veterinary or animal studies scientometrics and the respective number of published papers.

Combinations of countries			No. of collaborative papers published (n = 57)
Australia	Brazil		1
Australia	Canada		1
Australia	Poland		1
Australia	United Kingdom		3
Belgium	Brazil		1
Belgium	Italy		1
Brazil	United Kingdom		1
Brazil	Itali		1
Brazil	Portugal		1
Brazil	United States of America		1
Brunei	Malaysia		1
Chile	Colombia		1
China	Japan		1
Chile	Norway		1
China	Sweden		1
China	United States of America		1
Croatia	United States of America		1
Egypt	Saudi Arabia		2
Finland	Russia		1
Indonesia	Malaysia		1
India	Pakistan		1
India	Russia		1
India	United States of America		1
Italy	Poland		1
Italy	Spain		2
Portugal	Spain		1
Spain	United States of America		2
Turkiye	United States of America		1
Philippines	Taiwan		1
Australia	China	United States of America	1
Benin	United Kingdom	United States of America	1
Brazil	Colombia	Cuba	1
Brazil	Italy	Poland	1
Canada	China	Iran	1
Chile	Ecuador	Spain	1
China	Egypt	Turkiye	1
China	Nigeria	Sudan	1
Costa-Rica	Ecuador	Spain	1

Dominican Republic	Spain	United States of America			1
France	Morocco	Tunisia			1
Germany	Iran	Korea			1
Italy	United Kingdom	United States of America			1
Spain	Uganda	United States of America			1
Brazil	Chile	Venezuela			1
Algeria	Estonia	Indonesia	Malaysia		1
China	Indonesia	Pakistan	Saudi Arabia		1
China	Korea	Pakistan	Spain		1
China	Pakistan	Saudi Arabia	United Arab Emirates		1
Germany	Indonesia	United Kingdom	Uruguay		1
Egypt	Germany	Pakistan	Taiwan		1
Australia	Canada	Czech Republic	Sweden	United States of America	1
Korea	Nigeria	Mexico	United Kingdom	United States of America	1

Table S6. The organizations (n = 306) of origin of published papers on veterinary or animal studies scientometrics and the respective number of published papers.

Scientific organization	Country [1]	No. of published papers (n = 163)
Universities (n = 240)		
North Carolina State University	USA	5
State University of Sao Paulo	BRA	5
Federal University of Parana	BRA	4
University of Lisbon	PRT	4
University of Naples Federico II	ITA	4
Cairo University	EGY	3
Complutense University of Madrid	ESP	3
Federal University of Lavras	BRA	3
Kafrelsheikh University	EGY	3
University Malaysia Terengganu	MYS	3
University of Extremadura	ESP	3
University of London	GBR	3
University of Queensland	AUS	3
University of Thessaly	GRC	3
Akdeniz University	TUR	2
An Najah National University	PSE	2
Autonomous University of Barcelona	ESP	2
Bangor University	GBR	2
Charles Sturt University	AUS	2
City University of Hong Kong	CHN	2
Cornell University	USA	2
Drexel University	USA	2
Durban University of Technology	ZAF	2
Federal Technological University of Parana	BRA	2
Federal University of Goias	BRA	2
Federal University of Rio Grande do Norte	BRA	2
Guru Angad Dev Veterinary & Animal Sciences University	IND	2
Hazara University	PAK	2
James Cook University	AUS	2
King Abdulaziz University	SAU	2
King Faisal University	SAU	2
Laurentian University	CAN	2
Minia University	EGY	2
National University Federico Villarreal	PER	2
Northeast Agricultural University	CHN	2
Ohio State University	USA	2
Oklahoma State University	USA	2
Oregon Health & Science University	USA	2

Pontifical Catholic University of Chile	CHL	2
Pontifical Catholic University of Goias	BRA	2
Sabzevar University of Medical Sciences	IRN	2
Scientific University of the South	PER	2
Shaanxi University of Technology	CHN	2
Sohag University	EGY	2
Southeast University - China	CHN	2
University Miguel Hernandez de Elche	ESP	2
University of Bio-Bio	CHL	2
University of Brasilia	BRA	2
University of Calgary	CAN	2
University of Campania “Luigi Vanvitelli”	ITA	2
University of Cordoba	ESP	2
University of Florence	ITA	2
University of Granada	ESP	2
University of Montreal	CAN	2
University of Murcia	ESP	2
University of New South Wales Sydney	AUS	2
University of Porto	PRT	2
University of Tras-os-Montes & Alto Douro	PRT	2
University of Turin	ITA	2
University of Zabol	IRN	2
Abbottabad University of Science and Technology	PAK	1
Agricultural University Krakow	POL	1
Airlangga University	IDN	1
Amazonian Regional University IKEAM	ECU	1
Asia University Taiwan	TWN	1
Ataturk University	TUR	1
Autonomous Metropolitan University - Mexico	MEX	1
Beijing Forestry University	CHN	1
Bharathidasan University	IND	1
Canakkale Onsekiz Mart University	TUR	1
Central South University	CHN	1
Cooperative University of Colombia	COL	1
Coventry University	GBR	1
Dalian Medical University	CHN	1
Darfour University College	SDN	1
Delta University for Science & Technology	EGY	1
Democritus University of Thrace	GRC	1
Distance State University	CRI	1
Federal Rural University of Pernambuco	BRA	1
Federal University of Alagoas	BRA	1
Federal University of Espirito Santo	BRA	1

Federal University of Ouro Preto	BRA	1
Federal University of Santa Catarina	BRA	1
Federal University of Sao Joao del-Rei	BRA	1
Federal University of Vales do Jequitinhonha e Mucuri	BRA	1
Frontera National University	CHL	1
Gadjah Mada University	IDN	1
Gaziantep University	TUR	1
Ghent University	BEL	1
Government College University Faisalabad	PAK	1
Government College University Lahore	PAK	1
Hallym University	KOR	1
Hamburg University of Applied Sciences	DEU	1
Handong Global University	KOR	1
Harran University	TUR	1
Hebrew University of Jerusalem	ISR	1
Iberoamerican University	DOM	1
Idaho State University	USA	1
Indiana State University	USA	1
International Islamic University Malaysia	MYS	1
Iowa State University	USA	1
IPB University	PRT	1
Islamic Azad University	IRN	1
Jagiellonian University	POL	1
Jinan University	CHN	1
Kansas State University	USA	1
Kaohsiung Medical University	TWN	1
Katholic University of Leuven	BEL	1
Khalifa University of Science & Technology	ARE	1
Konkuk University	KOR	1
Landmark University	NGA	1
Manchester Metropolitan University	GBR	1
Mazandaran University Medical Sciences	IRN	1
Memorial University Newfoundland	CAN	1
Millersville University of Pennsylvania	USA	1
Mississippi State University	USA	1
Monash University	AUS	1
Muhammad Nawaz Shareef University of Agriculture	PAK	1
Nantes University	FRA	1
National Pingtung University Science & Technology	TWN	1
National Sun Yat Sen University	CHN	1
National Taiwan University	TWN	1
National University Mayor de San Marcos	PER	1
National University Micaela Bastidas De Apurimac	PER	1

National University of Altiplano	PER	1
National University of Mar del Plata	ARG	1
National University of Tachira	VEN	1
New Mexico University	USA	1
Nicolaus Copernicus University	POL	1
Nile University of Nigeria	NGA	1
Northwest A&F University	CHN	1
Northwest University Xi'an	CHN	1
Parthenope University Naples	ITA	1
Payame Noor University	IRN	1
Pirogov Medical University	RUS	1
Pontifical Catholic University of Minas Gerais	BRA	1
Pontifical Catholic University of Parana	BRA	1
Pontifical University of Javeriana	COL	1
Public University of Navarra	ESP	1
Purdue University	USA	1
Qilu University of Technology	CHN	1
Qujing Normal University	CHN	1
Rhodes University	ZAF	1
Rice University	USA	1
Sechenov First Moscow State Medical University	RUS	1
Sejong University	KOR	1
Shandong Agricultural University	CHN	1
Shanghai Jiao Tong University	CHN	1
Shanghai Ocean University	CHN	1
Sichuan University	CHN	1
Southern University of Science & Technology	CHN	1
Sri Vankateshwar University	IND	1
State University of Ponta Grossa	BRA	1
Suez Canal University	EGY	1
Sultan Sharif Ali Islamic University	BRN	1
Swedish University of Agricultural Sciences	SWE	1
Taipei Medical University	TWN	1
Tarbiat Modares University	IRN	1
Technical University of Manabi	ECU	1
Texas A&M University	USA	1
Tokyo Medical & Dental University	JPN	1
Tunku Abdul Rahman University of Management and Technology	MYS	1
United Arab Emirate University	ARE	1
University Andres Bello	CHL	1
University Badji Mokhtar - Annaba	DZA	1
University CEU Cardenal Herrera	ESP	1
University Feevale	BRA	1

University of Abomey Calavi	BEN	1
University of Agriculture, Abeokuta	NGA	1
University of Amsterdam	NLD	1
University of Antioquia	COL	1
University of Aveiro	PRT	1
University of Bari Aldo Moro	ITA	1
University of Basel	CHE	1
University of Basque Country	ESP	1
University of Benin	BEN	1
University of Birjand	IRN	1
University of Bologna	ITA	1
University of Bonn	DEU	1
University of British Columbia	CAN	1
University of Cadiz	ESP	1
University of California Davis	USA	1
University of Castilla-La Mancha	ESP	1
University of Chile	CHL	1
University of Chinese Academy of Sciences	CHN	1
University of Cordoba (Colombia)	COL	1
University of Edinburgh	GBR	1
University of Florida	USA	1
University of Guelph	CAN	1
University of Hamburg	DEU	1
University of Haripur	PAK	1
University of Ilorin	NGA	1
University of Indonesia	IDN	1
University of La Frontera	CHL	1
University of la Republica, Uruguay	URU	1
University of Leiden	NLD	1
University of Medical Sciences of La Habana	CUB	1
University of Melbourne	AUS	1
University of Minnesota	USA	1
University of Missouri	USA	1
University of Nottingham	GBR	1
University of Oklahoma	USA	1
University of Oslo	NOR	1
University of Oulu	FIN	1
University of Oxford	GBR	1
University of Padua	ITA	1
University of Parma	ITA	1
University of Puerto Rico	USA	1
University of Salford	GBR	1
University of Santiago de Compostela	ESP	1

University of Sao Paulo	BRA	1
University of Saskatchewan	CAN	1
University of Sassari	ITA	1
University of South Bohemia Ceske Budejovice	CZE	1
University of Split	HRV	1
University of Tartu	EST	1
University of Technology Sydney	AUS	1
University of Texas	USA	1
University of the Arctic	FIN	1
University of the Philippines Los Banos	PHL	1
University of the State of Mato Grosso	BRA	1
University of the State of Santa Catarina	BRA	1
University of Tsukuba	JPN	1
University of Valencia	ESP	1
University of Valle	COL	1
University of Wisconsin Madison	USA	1
University Paris Cite	FRA	1
University Ricardo Palma	PER	1
University Rovira and Virgili	ESP	1
University San Ignacio de Loyola	PER	1
University Science & Technology Houari Boumediene	DZA	1
University Sultan Zainal Abidin	MYS	1
University Syiah Kuala	IDN	1
University Teknologi MARA	MYS	1
Uppsala University	SWE	1
Utrecht University	NLD	1
Wuhan University	CHN	1
Yangzhou University	CHN	1
Yuzuncu Yil University	TUR	1
Zhejiang University	CHN	1
Other organizations (*n* = 66)		
Indian Council of Agricultural Research	IND	7
Council of Scientific & Industrial Research - India	IND	3
Chinese Academy of Sciences	CHN	3
National Institut for Research in Agriculture and the Environment	FRA	3
National Agency for Safety of Food and the Work Environment	FRA	3
Birla Institute of Technology & Science Pilani	IND	2
Research Institute of Zabol	IRN	2
Russian Academy of Sciences	RUS	2
Academic Center for Education, Culture & Research	IRN	1
Academy of Athens	GRC	1
Agronomic and Veterinary Institute Hassan II	MAR	1
American College of Veterinary Pharmacists	USA	1

Amrita Vishwa Vidyapeetham	IND	1
Basque Foundation for Science	ESP	1
Born Free Foundation	USA	1
Chinese Academy of Agricultural Sciences	CHN	1
Chinese Center for Disease Control & Prevention	CHN	1
Consejo Nacional de Investigaciones Cientificas y Tecnicas	ARG	1
Consiglio per la Ricerca in Agricoltura e L'analisi Dell'economia Agraria	ITA	1
Crayfish Aqua Venture	MYS	1
Empresa Brasileira de Pesquisa Agropecuaria	BRA	1
Federal Service for Supervision of Consumer Rights Protection and Human Well–being	RUS	1
Finnish Food Authority	FIN	1
Fish Farming and Technology Institute	EGY	1
Fisheries & Oceans Canada	CAN	1
Foundation for Research Development	ZAF	1
FRAME – United Kingdom	GBR	1
Friedrich Loeffler Institute	DEU	1
Fundación Ciencia & Vida Chile	CHL	1
Georgia Institute of Technology	USA	1
Government of Newfoundland and Labrador	CAN	1
Grupo Empresarial LABIOFAM	CUB	1
Higher Council of Scientific Investigations	ESP	1
Indian Institute of Technology Ropar	IND	1
Institut de Recerca de la Sida - IrsiCaixa	ESP	1
Institut de Recherche pour le Developpement	FRA	1
Institut Français du cheval et de l'équitation	FRA	1
Institute of Agrifood Research and Technology	ESP	1
Institution de la Recherche et de l'enseignement Superieur Agricoles	TUN	1
Instituto de Energías Renovables	MEX	1
Instituto de Investigación Hospital 12 de Octubre	ESP	1
Instituto de Investigacion Sanitaria y Biomedica de Alicante	ESP	1
Instituto Federal do Norte de Minas Gerais	BRA	1
Instituto Federal do Rio Grande do Norte	BRA	1
Instituto Federal Goiano	BRA	1
Instituto Nacional de Neurología y Neurocirugía	MEX	1
JSS Academy of Higher Education & Research	IND	1
Ministère de l'Agriculture Maroc	MAR	1
Ministry of Agriculture & Rural Affairs	CHN	1
Ministry of Health Italy	ITA	1
Museu Paraense Emilio Goeldi	BRA	1
National Agricultural Research Organization	UGA	1
National Center for International Research on Tropical Disease	CHN	1
National Institute of Oceanography & Fisheries	EGY	1
National Research & Innovation Agency of Indonesia	IDN	1

Natural Environment Research Council	GBR	1
Private Dental Office Dentilus AG	CHE	1
Private Dental Office Grimm Zahnärzte	CHE	1
Royal Netherlands Academy of Arts & Sciences	NLD	1
SciBiolMed.Org	IND	1
Scientific and Technical Research Center on Arid Regions Algeria	DZA	1
Secretario de Agricultura y Desarollo Rural	MEX	1
The Chinese People's Liberation Army	CHN	1
VetPharm Consulting	USA	1
World Health Organization	United Nations	1
You Guan Yi Biotechnology Company	TWN	1

[1] Abbreviations of country names according to the International Naming Convention ISO 3166 [International Organization for Standardization 2024].

Table S7. The databases ($n = 40$) used for record search in published papers on veterinary or animal studies scientometrics and the respective number of published papers, in which their use was reported.

Database used for record search	No. of published papers that used database
Web of Science	105
Scopus	60
PubMed	21
Google Scholar	15
ScienceDirect	12
Springer	7
Scielo	7
Indianjournals.com	6
J-Gate	6
CAB Abstracts	3
Dimensions	3
China National Knowledge Infrastructure	2
Embase	2
Agricola	1
Alicia-CONCYTEC-Perú	1
Animal Study Registry	1
BASE	1
Biological Abstracts	1
Biomed Central	1
Brazilian Bibliography of Dentistry	1
Brazilian Open Access Scientific Publications Portal (OASISbr)	1
Consortium of e-Resources in Agriculture	1
Current Contents	1
Deepbone	1
Directory of Open Access Journals and Abstracts Database of Reviews of Effects	1
Dissertation Abstracts International	1
Google	1
Grey Literature in Europe (Open Grey)	1
International Pharmaceutical Abstracts	1
Latin American and Caribbean Health Sciences Literature	1
LILACS	1
Microsoft Academic	1
ResearchGate	1
Science Literature Indicators Database	1
Veterinary Bulletin	1
VIP Chinese Journal Database (VIP)	1
Wanfang Data	1
Wildlife and Ecology Studies	1
WorldCat Library Catalog	1

Zoological Records	1

Table S8. The databases used most frequently (n = 9) for record search in published papers on veterinary or animal studies scientometrics and the year of publication of respective papers that reported use of database.

Database used for record search	Median year of publication (interquartile range) of papers
Google Scholar	2022 (1.5)
Indianjournals.com	2023 (2)
J-Gate	2023 (2)
PubMed	2022 (2)
Scielo	2022 (3)
ScienceDirect	2022 (2)
Scopus	2022 (2)
Springer	2022 (1.5)
Web of Science	2022 (3)

Table S9. Frequency of published papers on veterinary or animal studies scientometrics, in accord with the country of origin of the papers and the database used for record search.

	Databases used								
Country	Google Scholar	Indianjournals.com	J-Gate	PubMed	Scielo	ScienceDirect	Scopus	Springer	Web of Science
AUS	0	0	0	2	0	0	3	0	9
BRA	2	0	0	2	5	3	15	0	14
CAN	0	0	0	0	0	0	1	0	6
CHL	0	0	0	1	0	0	3	0	4
CHN	1	0	0	1	0	0	2	0	18
EGY	1	0	0	0	0	0	0	0	4
FRA	0	0	0	0	0	0	1	0	3
IND	7	6	6	7	0	7	10	7	2
IRN	0	0	0	0	0	0	0	0	5
ITA	0	0	0	0	0	1	3	0	10
PAK	0	0	0	0	0	0	1	0	4
PRT	1	0	0	1	0	0	2	0	5
ESP	1	0	0	3	1	1	6	0	13
TUR	1	0	0	0	0	0	1	0	4
GBR	0	0	0	1	1	1	4	0	9
USA	2	1	1	6	1	2	8	1	14

[1] Abbreviations of country names according to the International Naming Convention ISO 3166 [International Organization for Standardization 2024].

Figure S2. The databases used most frequently (n = 9) for record search for published papers on veterinary or animal studies scientometrics and the timespan of searches in respective published papers.

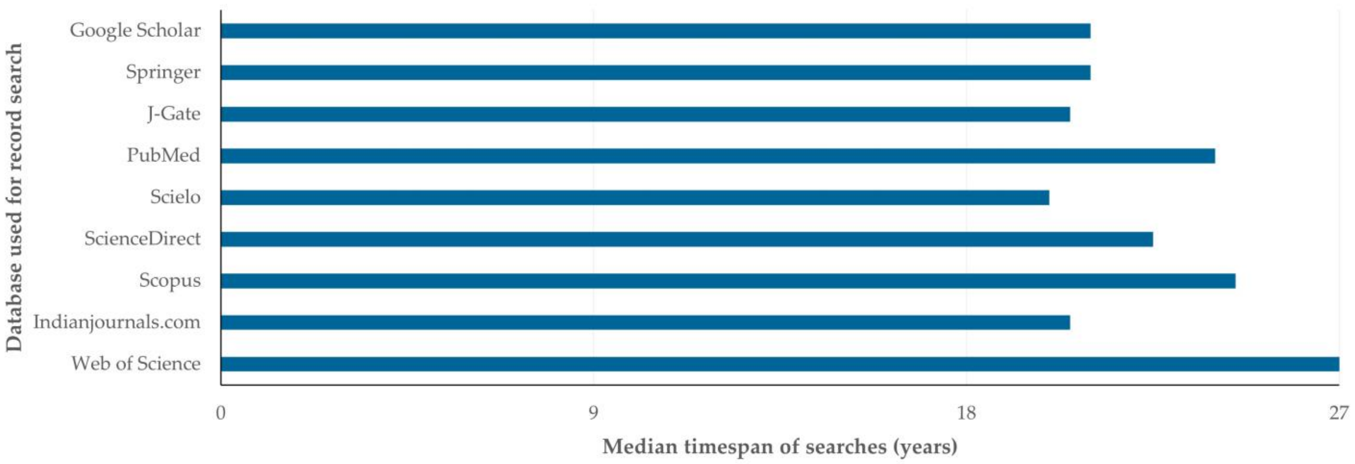

Table S10. The databases used most frequently (n = 9) for record search in published papers on veterinary or animal studies scientometrics and the number of records in respective studies.

Database used for record search	Median number (interquartile range) of records
Google Scholar	184 (207.5)
Indianjournals.com	217 (237)
J-Gate	217 (237)
PubMed	184 (529)
Scielo	145 (298.5)
ScienceDirect	164.5 (381)
Scopus	523 (2854.5)
Springer	184 (261.5)
Web of Science	1072 (5650.5)

Table S11. The journals (n = 106) in which papers on veterinary or animal studies scientometrics were published, and the respective number of published papers.

Journal	No. of published papers (n = 163)
Animals	21
Scientometrics	8
Frontiers in Veterinary Science	7
Pathogens	4
Poultry Science	3
Veterinary Sciences	3
Anthrozoos	2
Conservation Biology	2
Ecological Informatics	2
Environmental Evidence	2
Facets	2
Frontiers in Ecology and Evolution	2
Indian Journal of Animal Sciences	2
INRA Productions Animales	2
International Journal of Environmental Research and Public Health	2
Italian Journal of Animal Science	2
Journal of the Medical Library Association	2
Revista de Investigaciones Veterinarias del Peru	2
Revista MVZ Cordoba	2
Transboundary and Emerging Diseases	2
Veterinary Anaesthesia and Analgesia	2
Veterinary World	2
Zoo Biology	2
Acta Parasitologica	1
Acta Radiologica	1
Agricultura Sociedad y Desarrollo	1
American Journal of Pharmaceutical Education	1
Animal Biodiversity and Conservation	1
Animal Cognition	1
Animal Welfare	1
Annals of Library and Information Studies	1
Applied Biochemistry and Microbiology	1
Aquaculture International	1
Aquatic Ecosystem Health & Management	1
Archives of Animal Breeding	1
Archives of Public Health	1
ATLA-Alternatives to Laboratory Animals	1
Biomed Research International	1
BMC Veterinary Research	1
Chilean Journal of Agricultural & Animal Sciences	1

College & Research Libraries	1
Critical Public Health	1
Cureus Journal of Medical Science	1
Current Research in Food Science	1
Current Research in Parasitology & Vector-Borne Diseases	1
Current Science	1
Dentistry Journal	1
Diseases	1
Ecological Questions	1
Environmental Science and Pollution Research	1
Environmental Toxicology and Pharmacology	1
Environments	1
European Journal of Therapeutics	1
Food and Waterborne Parasitology	1
Frontiers in Nutrition	1
Frontiers in Physiology	1
Frontiers in Public Health	1
Helgoland Marine Research	1
Heliyon	1
Historical Biology	1
Human Vaccines & Immunotherapeutics	1
Information	1
International Journal of Biometeorology	1
International Journal of Tropical Insect Science	1
Iranian Journal of Parasitology	1
Journal of Academic Librarianship	1
Journal of Animal and Veterinary Advances	1
Journal of Animal Breeding and Genetics	1
Journal of Animal Science	1
Journal of Coastal Conservation	1
Journal of Ecotourism	1
Journal of Environmental Sciences	1
Journal of Health Population and Nutrition	1
Journal of Osseointegration	1
Journal of Pharmacy & Pharmacognosy Research	1
Journal of Veterinary Medical Education	1
Journal of Young Pharmacists	1
Laboratory Animals	1
Livestock Science	1
Neotropical Biology and Conservation	1
One Health	1
Parasites & Vectors	1
Pesquisa Veterinaria Brasileira	1

Plos ONE	1
Research in Veterinary Science	1
Revista Chilena de Historia Natural	1
Revista de Biologia Tropical	1
Revista de Salud Ambiental	1
Science and Public Policy	1
Science Technology and Society	1
Semina-Ciencias Agrarias	1
Sensors	1
South African Journal of Botany	1
Sustainability	1
Sustainable Materials and Technologies	1
Transinformacao	1
Trends in Food Science & Technology	1
Tropical Animal Health and Production	1
Turkish Journal of Veterinary & Animal Sciences	1
Veterinary Ophthalmology	1
Veterinary Parasitology- Regional Studies and Reports	1
Veterinary Quarterly	1
Veterinary Research	1
Vigilancia Sanitaria em Debate-Sociedade Ciencia & Tecnologia	1
Viruses	1
Zebrafish	1

Table S12. Frequency of published papers on veterinary or animal studies scientometrics, in accord with the country [1] of origin of the papers and the journal [2] in which they were published.

	Journal in which paper was published																						
Country	*Animals*	*Anthrozoos*	*Conserv Biol*	*Ecol Inform*	*Environ Evid*	*Facets*	*Front Ecol Evol*	*Front Vet Sci*	*Ind J Anim Sci*	*INRA Prod Anim*	*Int J Env Res Pub Heal*	*It J Anim Sci*	*J Med Libr Assoc*	*Pathogens*	*Poultry Sci*	*Rev Investig Vet Peru*	*Rev MVZ Cordoba*	*Scientometrics*	*Transbound Emerg Dis*	*Vet Anaes Analg*	*Vet Sci*	*Vet World*	*Zoo Biol*
AUS	2	0	1	0	2	1	0	1	0	0	0	0	0	1	0	0	0	0	0	0	0	0	0
BRA	4	0	0	0	0	0	0	0	0	0	0	0	0	0	0	0	0	1	0	0	1	0	1
CAN	0	0	1	0	1	2	0	0	0	0	0	0	1	0	1	0	0	0	0	2	0	0	0
CHL	0	0	0	0	0	0	0	0	0	0	0	0	0	0	0	0	0	0	0	0	0	0	0
CHN	3	0	0	2	0	0	0	2	0	0	0	0	0	1	1	0	0	0	0	0	1	0	0
EGY	0	0	0	0	0	0	0	2	0	0	0	0	0	0	0	0	0	0	0	0	0	0	0
FRA	0	0	0	0	0	0	0	0	0	2	0	0	0	0	0	0	0	1	0	0	0	0	0
IND	1	0	0	0	0	0	0	0	0	0	0	0	0	0	3	0	0	0	0	0	0	0	0
IRN	8	0	0	0	0	0	0	0	0	0	0	2	0	0	0	0	0	0	0	0	0	0	0
ITA	0	0	0	0	0	0	0	0	2	0	1	0	0	1	0	0	0	2	0	0	0	1	0
PAK	1	0	0	0	0	0	0	0	1	0	0	0	0	0	0	0	0	0	0	0	0	0	0
PRT	0	0	0	0	0	0	0	0	0	0	0	0	0	0	0	0	0	0	2	0	0	0	0
ESP	4	0	0	0	0	0	0	1	0	0	0	0	0	0	0	0	0	0	0	0	0	0	0
TUR	0	0	0	0	0	0	0	0	0	0	0	0	0	0	0	0	0	0	0	0	0	0	0
GBR	1	0	1	0	0	0	1	1	0	0	1	0	0	0	0	0	0	1	0	0	0	0	0
USA	1	1	0	0	1	0	2	2	0	0	0	0	1	2	0	0	0	1	0	0	1	0	1

[1] Abbreviations of country names according to the International Naming Convention ISO 3166 [International Organization for Standardization 2024].

[2] Abbreviations of names of journals are as follows: *Animals*: *Animals*, *Anthrozoos*: *Anthrozoos*, *Conserv Biol*: *Conservation Biology*, *Ecol Inform*: *Ecological Informatics*, *Environ Evid*: *Environmental Evidence*, *Facets*: *Facets*, *Front Ecol Evol*: *Frontiers in Ecology and Evolution*, *Front Vet Sci*: *Frontiers in Veterinary Science*, *Ind J Anim Sci*: *Indian Journal of Animal Science*, *INRA Prod Anim*: *INRA Productions Animals*, *Int J Env Res Pub Heal*: *International Journal of Environmental Research and Public Health*, *It J Anim Sci*: *Italian Journal of Animal Science*, *J Med Libr Assoc*: *Journal of the Medical Library Association*, *Pathogens*: *Pathogens*, *Poultry*

Sci: *Poultry Science*, *Rev Investig Vet Peru*: *Revista de Investigaciones Veterinarias del Peru*, *Rev MVZ Cordoba*: Revista MVZ Cordoba, *Scientometrics*: *Scientometrics*, *Transbound Emerg Dis*: *Transboundary and Emerging Diseases*, *Vet Anaes Analg*: *Veterinary Anaesthesia and Analgesia*, *Vet Sci*: *Veterinary Science*, *Vet World*: *Veterinary World*, *Zoo Biol*: *Zoo Biology*.

Table S13. The Web of Science categories (n = 56) in which journals with published papers on veterinary or animal studies scientometrics were classified, and the respective number of journals and of published papers in these journals.

Web of Science category	No. of journals in category	No. of published papers in journal	Published papers per journal
Veterinary sciences	26	61	2.3
Environmental sciences	13	16	1.2
Agriculture, dairy & animal science	11	36	3.3
Public environmental & occupational health	7	8	1.1
Zoology	7	8	1.1
Ecology	6	9	1.5
Information science & library science	6	14	2.3
Parasitology	6	6	1.0
Biodiversity conservation	4	5	1.3
Pharmacology & pharmacy	4	4	1.0
Biotechnology and applied microbiology	3	3	1.0
Marine & freshwater biology	3	3	1.0
Medicine, research & experimental	3	3	1.0
Multidisiplinary sciences	3	5	1.7
Dentistry, oral surgery & medicine	2	2	1.0
Education, scientific disciplines	2	2	1.0
Food science & technology	2	2	1.0
Green and sustainable science & technology	2	2	1.0
Infectious diseases	2	3	1.5
Management	2	2	1.0
Medicine, general & internal	2	2	1.0
Microbiology	2	5	2.5
Physiology	2	2	1.0
Agriculture, enonomics & policy	1	1	1.0
Agriculture, multidisiplinary	1	1	1.0
Agronomy	1	1	1.0
Behavioural sciences	1	1	1.0
Biology	1	1	1.0
Biophysics	1	1	1.0
Chemistry, analytical	1	1	1.0
Computer science, information systems	1	1	1.0
Computer science, interdisciplinary applications	1	8	8.0
Developmental biology	1	1	1.0
Energy & fuels	1	1	1.0
Engineering, electrical & electronic	1	1	1.0
Entomology	1	1	1.0
Fisheries	1	1	1.0
Health care sciences & services	1	1	1.0
Hospitality, leisure, sport & tourism	1	1	1.0

Immunology	1	1	1.0
Instruments & instrumentation	1	1	1.0
Materials science, multidisiplinary	1	1	1.0
Meteorology & atmospheric sciences	1	1	1.0
Nuclear medicine & medical imaging	1	1	1.0
Nutrition & dietetics	1	1	1.0
Oceanography	1	1	1.0
Paleontology	1	1	1.0
Plant sciences	1	1	1.0
Public administration	1	1	1.0
Radiology	1	1	1.0
Social sciences, biomedical	1	1	1.0
Sociology	1	2	2.0
Toxicology	1	1	1.0
Tropical medicine	1	1	1.0
Virology	1	1	1.0

Table S14. The topics (n = 88) assessed in work reported in published papers on veterinary or animal studies scientometrics and the respective number of published papers.

Topic of assessment	No. of published papers in which topic was assessed
Country-related scientometrics (details in Table S15)	16
Specific journal-related scientometrics (details in Table S16)	10
Animal species-related scientometrics (details in Table S17)	10
Animal welfare	10
Genetic improvement	6
Animal ethology	5
Mastitis	5
Conservation plan	4
Antibiotic resistance	3
Gastrointestinal parasitism	3
Heat stress	3
Human-animal interactions	3
Veterinary education	3
Epilepsy	2
Infections	2
Precision livestock farming	2
Tuberculosis	2
Zoo archaeology - palaeontology	2
African swine fever	1
Anaesthesia & analgesia	1
Animal model	1
Animal waste	1
Anticoccidials	1
Antileishmanial drugs	1
Antiparasitic ecotoxicology	1
Avian leucosis	1
Campylobacter infections	1
β-casein	1
Cattle meat supply chain	1
Chelates in animal nutrition	1
Chemical residues	1
Chlorella in aquaculture	1
Circovirus infection	1
Climate change & zoonoses	1
Covid-19 animal models	1
Cysticercosis	1
Dicrocoelium dendriticum infection	1
Dental care	1
Dental implants	1

Diagnostic imaging	1
Digital & precision technologies	1
Echinococcosis	1
Echolocation	1
Embryos	1
Equine infectious anaemia	1
Fasciolosis	1
Feed production	1
Foot health	1
Forage cactus feeding	1
Health & welfare	1
Health management	1
Housing	1
Humoral immunity	1
IgY	1
Influenza	1
Intestinal health	1
Intracranial aneyrysm	1
Kosher meat production	1
Leishmaniosis treatment	1
Mapping in veterinary research	1
Milk casein	1
Neospora infection	1
Non-conventional therapies	1
Nutritional values	1
One health research	1
Ophthalmology	1
Organic production	1
Parasites	1
Peste des petis ruminants	1
Pharmacy	1
PRRS	1
Q fever	1
Rabies vaccination	1
Ranavirus infections	1
Reproduction	1
Reproductive biotechnology	1
Sheep- & goat-pox	1
Swine diseases	1
Tick infestation & tick-borne diseases	1
Toxicology	1
Toxoplasma infection	1
Traumatology	1

Venom	1
Veterinary entomology	1
Veterinary vaccines	1
Vibrio alginolyticus infections	1
Viral infections	1
Zoonoses	1

Table S15. The countries referred to in work reported in published papers on veterinary or animal studies scientometrics in the topic of assessment of country-related scientometrics and the respective number of published papers.

Topic of assessment	No. of published papers in which coutnry was referred to
India	4
Brazil	2
The Netherlands	2
Global	1
Algeria	1
Chile	1
Colombia	1
France	1
Greece	1
Morocco	1
South Africa	1
Tunisia	1

Table S16. The journals referred to in work reported in published papers on veterinary or animal studies scientometrics in the topic of assessment of specific journal-related scientometrics and the respective number of published papers.

Topic of assessment	No. of published papers in which journal was referred to
all journals in the Web of Science category 'Veterinary Sciences'	1
50 journals in the Web of Science category 'Agriculture, Dairy & Animal Science' [1]	1
Alternatives to Laboratory Animals	1
American Journal of Veterinary Research	1
American Journal of Veterinary Research, Compendium of Continuing Education for the Practicing Veterinarian, Journal of the American Animal Hospital Association, Journal of the American Veterinary Medical Association, Journal of Small Animal Practice, Research in Veterinary Science, Theriogenology, Veterinary Clinics of North America Small Animal Practice, Veterinary Medicine, Veterinary Microbiology, Veterinary Parasitology, The Veterinary Record	1
Annual Review of Animal Biosciences, Genetics Selection Evolution, Journal of Animal Science and Biotechnology, Journal of Dairy Science, Poultry Science	1
Journal of Veterinary Medical Education	1
Revista MVZ Cordoba	1
Veterinary Anaesthesia and Analgesia	1
Zoo Biology	1

[1] *Acta Agriculturae Scandinavica Section A—Animal Science, Animal, Animal Biotechnology, Animal Feed Science and Technology, Animal Genetics, Animal Nutrition and Feed Technology, Animal Reproduction Science, Animal Science Journal, Animal Science Papers and Reports, Annals of Animal Science, Applied Animal Behaviour Science, Archiv fur Geflugelkunde, Archives of Animal Breeding, Archives of Animal Nutrition, Australian Journal of Dairy Technology, Avian and Poultry Biology Reviews, Avian Biology Research, Brazilian Journal of Poultry Science, British Poultry Science Journal, Canadian Journal of Animal Science, Cuban Journal of Agricultural Science, Czech Journal of Animal Science, Domestic Animal Endocrinology, Genetics Selection Evolution, Indian Journal of Animal Sciences, Italian Journal of Animal Science, Journal of Animal and Feed Sciences, Journal of Animal Breeding and Genetics, Journal of Animal Physiology and Animal Nutrition, Journal of Animal Science, Journal of Applied Animal Research, Journal of Applied Poultry Research, Journal of Dairy Research, Journal of Dairy Science, Journal of Reproduction and Development, Livestock Science, Mljekarstvo, Poultry Science, Productions Animales, Reproduction in Domestic Animals, Revista brasileira de zootecnia—Brazilian Journal of Animal Science, Revista MVZ Cordoba, Sabrao Journal of Breeding and Genetics, Small Ruminant Research, South African Journal of Animal Science, Tecnica Pecuaria en Mexico, Tropical Grasslands, World Rabbit Science, Worlds Poultry Science Journal, Zuchtungskunde.*

Table S17. The animal species referred to in work reported in published papers on veterinary or animal studies scientometrics in the topic of assessment of specific animal species-related scientometrics and the respective number of published papers.

Topic of assessment	No. of published papers in which the animal species was referred to
Camels	2
Crabs	1
Crayfish	1
Elasmobrachs	1
Horses	1
Lions	1
Octopuses	1
Poultry	1
Zoo and aquaria animals	1

Table S18. The topics assessed most frequently ($n = 7$) in published papers on veterinary or animal studies scientometrics and the year of publication of respective papers.

Topic of assessment	Median year of publication (interquartile range) of papers
Country-related scientometrics	2018 (11)
Specific journal-related scientometrics	2011 (12)
Animal species-related scientometrics	2021 (7)
Animal welfare	2021 (3)
Genetic improvement	2023 (6)
Animal ethology	2023 (2)
Mastitis	2021 (4)

Table S19. The topics assessed most frequently (*n* = 7) in published papers on veterinary or animal studies scientometrics and the number of records in studies reported in respective papers.

Topic of assessment	Median number (interquartile range) of records in papers
Country-related scientometrics	6464.5 (12954)
Specific journal-related scientometrics	1615.5 (16439)
Animal species-related scientometrics	6361.5 (5176)
Animal welfare	989.5 (3462)
Genetic improvement	3100 (5423)
Animal ethology	1525 (1456)
Mastitis	250 (336)

Table S20. The specific animal species (n = 38) included in studies reported in papers on veterinary or animal studies scientometrics and the respective number of published papers.

Animal species referred to	No. of published papers (n = 92)
Cattle	14
Poultry	12
Pigs	9
Sheep	9
Wildlife	9
Bees	6
Horses	6
Goats	5
Livestock	5
Aquatic animals	4
Dogs	4
Fish	4
Camels	3
Ruminants	3
Zoo animals	3
Buffaloes	2
Laboratory animals	2
Zebrafish	2
Bats	1
Bivalves	1
Cats	1
Crabs	1
Crayfish	1
Ferrets	1
Food-producing animals	1
Frogs	1
Lions	1
Marine animals	1
Mice	1
Octopus	1
Penguins	1
Pigeons	1
Rats	1
River dolphins	1
Sharks	1
Snakes	1
Sturgeon	1
Terrestrial animals	1

Figure S3. Box and whisker plot of number of records in papers on veterinary or animal studies scientometrics, in accord with the most frequently referred to specific animal species.

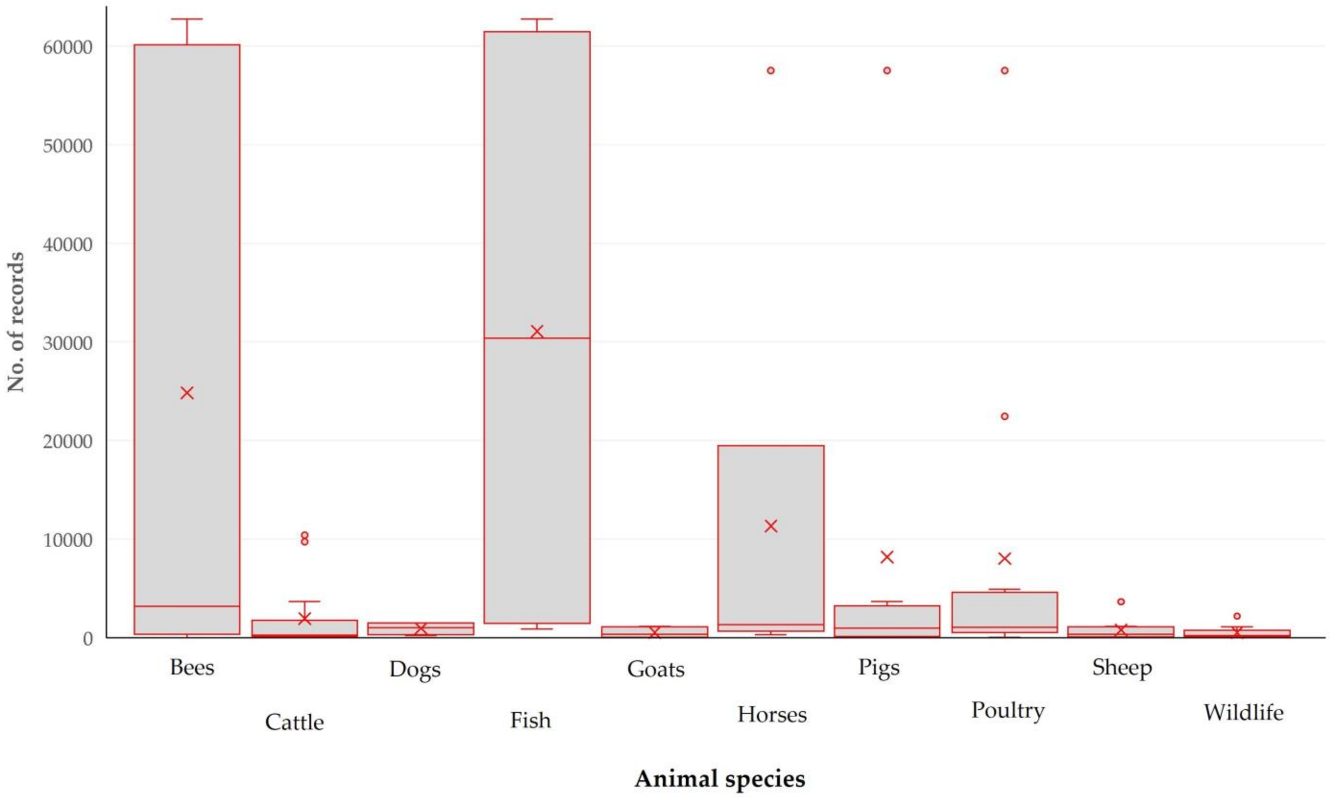

Table S21. The keywords ($n = 517$) in published papers on veterinary or animal studies scientometrics and the respective number of occurrences in the papers.

Keywords	No. of occurrences ($n = 791$)
Bibliometrics	45
Bibliometric analysis(es)	32
Scientometric(s)	17
VOS viewer	11
Meta-analysis	9
Animal welfare	8
One Health	8
Bibliometric review	6
Cattle	6
CiteSpace	6
Sheep	6
Systematic review	6
Bibliometrix	5
Research	5
Research trend(s)	5
Veterinary medicine	5
Animal model(s)	4
Aquaculture	4
Citation	4
Citation analysis	4
India	4
Prevalence	4
Publications	4
Web of science	4
Animals	3
Animal wellbeing	3
Author(s)	3
Camel(s)	3
China	3
Conservation	3
Gastrointestinal parasite(s)	3
h-index	3
International collaboration(s)	3
Journal impact factor	3
Mastitis	3
Meta-research	3
Pig(s)	3
Poultry	3
Precision livestock farming	3
Publication	3

Science mapping	3
Scopus	3
Sustainability	3
Veterinary	3
A2 milk	2
Africa	2
Agriculture	2
Animal science	2
Animal tuberculosis	2
Aqua feed	2
Asia	2
BCM-7	2
Beta-casein	2
BibliometrixR	2
Bibliometry	2
Biometeorology	2
Bovine	2
Chicken(s)	2
Citations	2
Climate change	2
COVID-19	2
Dairy cow(s)	2
Dog(s)	2
E. coli	2
Education	2
Epilepsy	2
Goat	2
Heat stress	2
Human-animal interaction	2
Impact factor	2
Infectious disease(s)	2
Journal	2
Livestock	2
Machine learning	2
Mammalia	2
Medicine	2
Milk proteins	2
Network analysis	2
Phylodynamics	2
Research hotspots	2
Scientific collaboration	2
Scientific production	2
Staphylococcus species	2

Subclinical mastitis	2
Vaccine	2
Veterinary education	2
Veterinary Science	2
VOS viewer software	2
Wildlife	2
Zoonosis	2
13-Casomorphin-7	1
A1 milk	1
Abortions	1
Abstracts and indexing	1
Academic age	1
Acupuncture	1
Adjuvant	1
Advisor	1
African	1
African swine fever	1
Agricultural research	1
Agricultural science	1
Agroforestry systems	1
Algeria	1
Allopurinol	1
Alternative therapies	1
Amphotericin	1
AMR	1
Analysis	1
Animal adaptation	1
Animal and dairy science journals	1
Animal behavior	1
Animal detection systems	1
Animal ethics	1
Animal experiments	1
Animal health and welfare	1
Animal mapping	1
Animal production	1
Animal research	1
Animal science literature	1
Animal studies	1
Animal welfare science	1
Anthropogenic impact	1
Anthrozoology	1
Antibacterial	1
Antibiotics	1

Anticoccidial	1
Antimicrobial alternatives	1
Antimicrobial resistance	1
Antimonials	1
Antiparasitic	1
Antiprotozoal agents	1
Applied zooarchaeology	1
Aquatic organism	1
Aquatic systems	1
Arctic	1
Argentina	1
ARRIVE	1
ASPA	1
Authorship	1
Avian leukosis viruses (ALVs)	1
Bacterial pathogens	1
Bat echolocation	1
Behavior	1
Behavior alecology	1
Behavioral science	1
Bias	1
Bibliometric-ALV	1
Bibliometric network	1
Bibliometric studies	1
Biblioshiny	1
Bioactive products	1
Biodiversity hotspots	1
Biological activities	1
Biological conversion	1
Bivalves	1
Black	1
Brasil	1
Breeding	1
Broilers	1
Buffaloes	1
Byproducts	1
Camel research	1
Campylobacter	1
Canine	1
Canine leishmaniasis	1
Cannibalism	1
Capripoxvirus	1
Carbon quantum dots	1

Caribbean region	1
Categorization	1
Centres of animal welfare	1
Cephalopods	1
Chelate	1
Chemotherapy	1
Chile	1
Chlorella	1
Chronological growth	1
Circovirus	1
Clients	1
Cognition	1
Collaboration index	1
Colombia	1
Communication	1
Complementary therapies	1
Compost barn	1
Compost-bedded pack barn	1
Conceptual framework	1
Conceptualization	1
CONSORT	1
Content analyses	1
Co-occurrence analysis	1
Co-reference	1
Corneal grafting	1
Corresponding role	1
Coupling	1
Coword analysis	1
Crab	1
Crayfish	1
Critical animal studies	1
Cross-disciplines	1
Cysticercosis	1
Cytotoxicity	1
Dairy cattle	1
Danio rerio	1
Database accuracy	1
Deep Bone database	1
Dental implants	1
Diagnosis	1
Diagnostic techniques	1
Dicrocoelium dendriticum	1
Digital dermatitis	1

Diversity	1
Dolphins	1
Drug monographs	1
Drug pollution	1
Echinococcosis	1
Echinococcus granulosus sensu lato	1
Echinococcus multilocularis	1
Economics	1
Ecotoxicology	1
Educational research methods	1
Educational scholarship	1
Egg yolk immunoglobulin (IgY)	1
Embryo	1
Emergent animal model	1
Emerging industry	1
Emerging topics	1
Endangered	1
Environmental effects	1
Environmental factors	1
Environmental health	1
Epidemiology	1
Equine anemia	1
Equine-assisted services	1
Essential oils	1
Evaluation	1
Evidence-based medicine	1
Evidence synthesis	1
Evolution	1
Ewe	1
Experimental model	1
Farm animal welfare	1
Fascioliasis	1
Feed	1
Feline	1
Financial support	1
First author ship	1
Fisheries	1
Fitness	1
Five domains model	1
Food	1
Food Composition	1
Food producing animals	1
Food Safety	1

Free stall	1
GAP analysis	1
Gen Bank	1
Gender	1
Genes	1
Genetic diversity and farmed animals	1
Genetic improvement	1
Geographic albias	1
Global	1
Global health	1
Global publications	1
Growth performance	1
Gut health	1
Hard release	1
Health and disease	1
Health hazards	1
Heat abatement	1
Homeopathy	1
Honey	1
Honeybees	1
Hoof disorder	1
Horses	1
Hotspots de biodiversidad	1
Hot topics	1
Human	1
Human animal studies	1
Human behavior	1
Human excreta	1
Humans	1
Human wildlife	1
Human wildlife conflict	1
Humoral immunity	1
Hydatidosis	1
ICAR	1
IgY technology	1
Immune responses	1
Immunoprophylaxis	1
Inclusion	1
Indian publications	1
Individual differences	1
Infanticide	1
Influenza variants	1
Information analysis	1

Insularity index	1
Intellectual structure	1
Intelligent systems	1
Interdisciplinary	1
Intergenerational inheritance	1
Internationalization	1
International research	1
Intracranial aneurysms (IAs)	1
Intramammary infection	1
Invasive	1
Invasive species	1
Journal evaluation	1
Journal *h* index	1
Journal of Clinical Periodontology	1
Journal of Periodontology	1
Keyword co-occurrence	1
Keyword co-occurrence analysis	1
Keywords	1
Kindling model	1
Knowledge gap	1
Knowledge visualisation	1
Kosher	1
Laboratory rodents	1
Laminitis	1
Law enforcement	1
LC/MS-MS	1
Leishmania infantum	1
Leishmaniasis treatment	1
Leishmania spp.	1
Listas rojas	1
Literacy skills	1
Literature analysis	1
Literature search	1
Livestock farming	1
Livestock manure	1
Livestock production	1
Livestock production system	1
Machine learning datasets	1
Mammary infection	1
Marco conceptual	1
Maternal aggression	1
Maternal effects	1
Meat	1

Medicinal plants	1
Metrics	1
Milking	1
Milk production	1
Miltefosine	1
Model species	1
Molecular epidemiology	1
Morocco	1
Multi-language	1
Nanomedicine	1
Natural shade	1
Neospora caninum	1
Network visualization	1
Neurocysticercosis	1
Neurotoxicology	1
Non humans	1
Nopalea	1
Nuclear transfer	1
Nutrients	1
Observational research	1
Opuntia	1
ORF5	1
Organic	1
Osseointegration	1
Ovine	1
Oxidative stress	1
Pain	1
Panthera leo	1
Parasite diseases	1
Paternal effects	1
Pathogen	1
Pathogenesis	1
Pathogenicity	1
Periodontal and peri-implant research	1
Pesticide	1
Pharmaceutical pollution	1
Pharmacy libraries	1
Phytotherapy	1
Pigeon circovirus	1
Popularising science(Source:EuroVoc)	1
Population management	1
Positive indicators	1
Posthumanism	1

PPR	1
Price index	1
Processing	1
Productivity index	1
Proteome	1
Proteomics	1
PRRSV	1
Psychoactive	1
Publication analysis	1
Publication output	1
Public health	1
Quality	1
Quality assessment	1
Rabies	1
Radiologists	1
Ranavirus	1
Rat	1
Rays	1
Rebound tonometry	1
Red lists	1
Reintroduction	1
Relative growth rate	1
Reporting guidelines	1
Representation	1
Reproduction	1
Reproductive biotechnology	1
Research analysis	1
Research assessment	1
Research coordination	1
Research frontiers	1
Research fronts	1
Research impact	1
Research mapping	1
Research networks	1
Research policy	1
Research priority	1
Research productivity	1
Research proliferation	1
Research publication	1
Research status	1
Residues	1
Review	1
Revisión sistemática	1

River-based	1
Sample size	1
Sb-V	1
Scholarly communication	1
Science	1
Science mapping analysis	1
Science-society dialogue	1
Scientific article	1
Scientific communication	1
Scientific journal	1
Scientific press	1
Scientific publications	1
Scientific trends	1
Scientometric analysis	1
Scientometric study	1
Scientometry	1
SCNT	1
Scoping review	1
Seizure	1
Semi-arid	1
Sensor	1
Serological techniques	1
Seroprevalence	1
Sharks	1
Sheep and goats	1
Slaughter process	1
Small animal	1
Small ruminants	1
Smart farming	1
Snake venom	1
Social network analysis	1
Soft release	1
Sole ulcer	1
Spheniscidae	1
SRBC	1
Staphylococcus	1
Stress	1
Student	1
Study of arthropods	1
Sturgeon	1
Subgroup analysis	1
Subject areas	1
Supply chain	1

Swine	1
Swine influenza virus	1
Taenia solium	1
Tamaño muestral	1
Technological surveillance	1
Technologies	1
Técnicas diagnósticas	1
Temperament	1
Text analytics	1
Text mining	1
Therapy	1
Thermochemical conversion	1
Tick	1
Tick-bornediseases	1
Tie-stall	1
Titanium dioxide nanoparticles	1
Toxic effect	1
Toxoplasma gondii	1
Trans-generation alinheritance	1
Treatment	1
Tunisia	1
Uganda	1
Undergraduate	1
Vector-borne diseases	1
Vertebrados	1
Vertebrate palaeontology	1
Vertebrates	1
Veterinarians	1
Veterinary dentistry	1
Veterinary pharmacy	1
Waste-to-energy	1
Web of science core collection	1
Web of Science Core Collection database	1
Welfare assessment	1
Well-being	1
Whole genome	1
Wild animal	1
Wildlife conservation	1
Wildlife health	1
Wildlife-vehicle collisions	1
Women	1
World	1
Young pigeon disease syndrome	1

Zebrafish	1
Zoo animal	1
Zoonotic bacteria	1
Zoos	1

Figure S4. Trendline of the progressive change of the median number of co-authors in papers on veterinary or animal studies scientometrics published yearly.

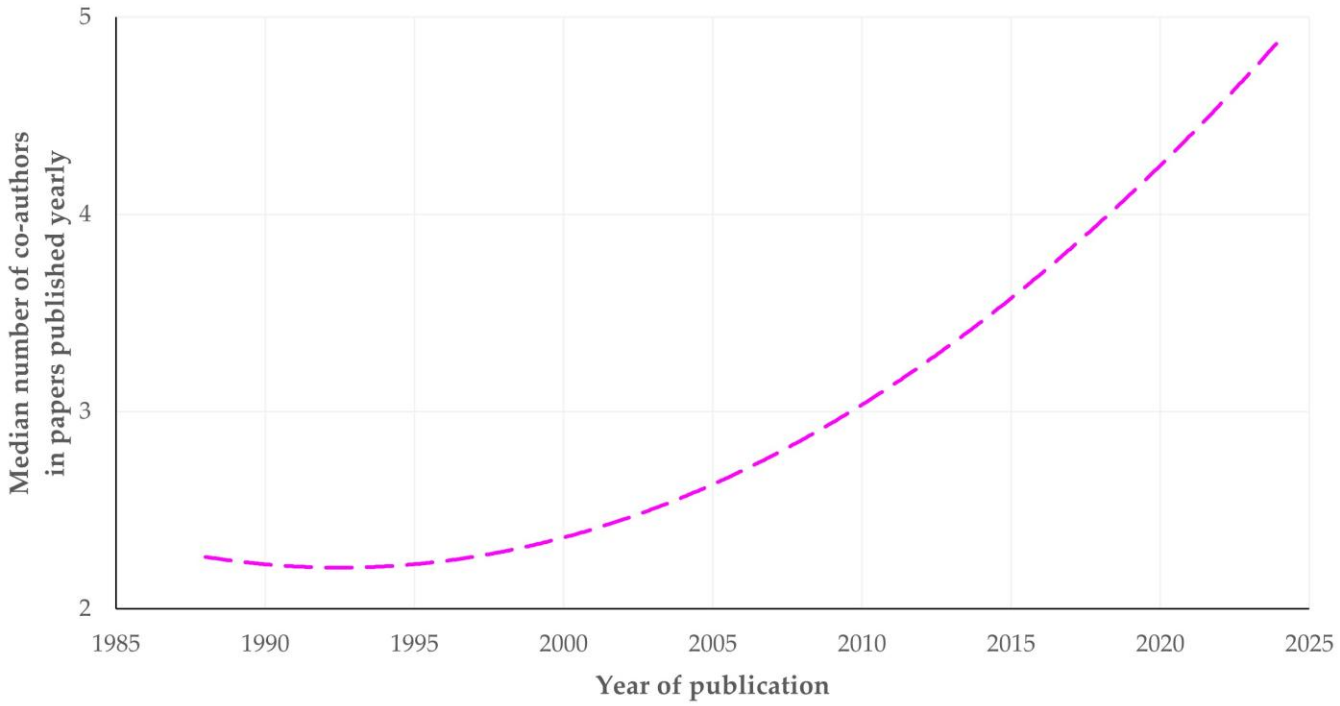

Figure S5. Box and whisker plot of number of co-authors in papers on veterinary or animal studies scientometrics, among the countries with most published papers.

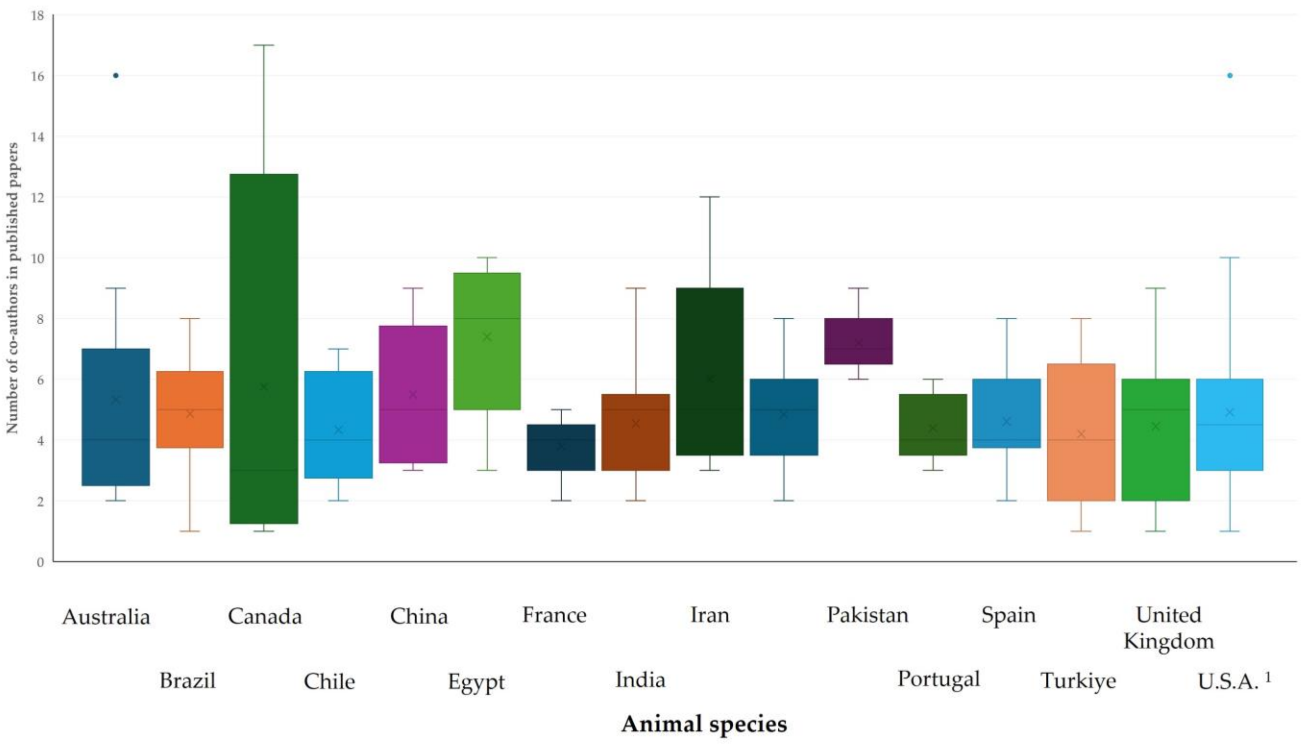

[1] United States of America.

Figure S6. The proportion of papers published under open access among the countries [1] with most published papers on veterinary or animal studies scientometrics.

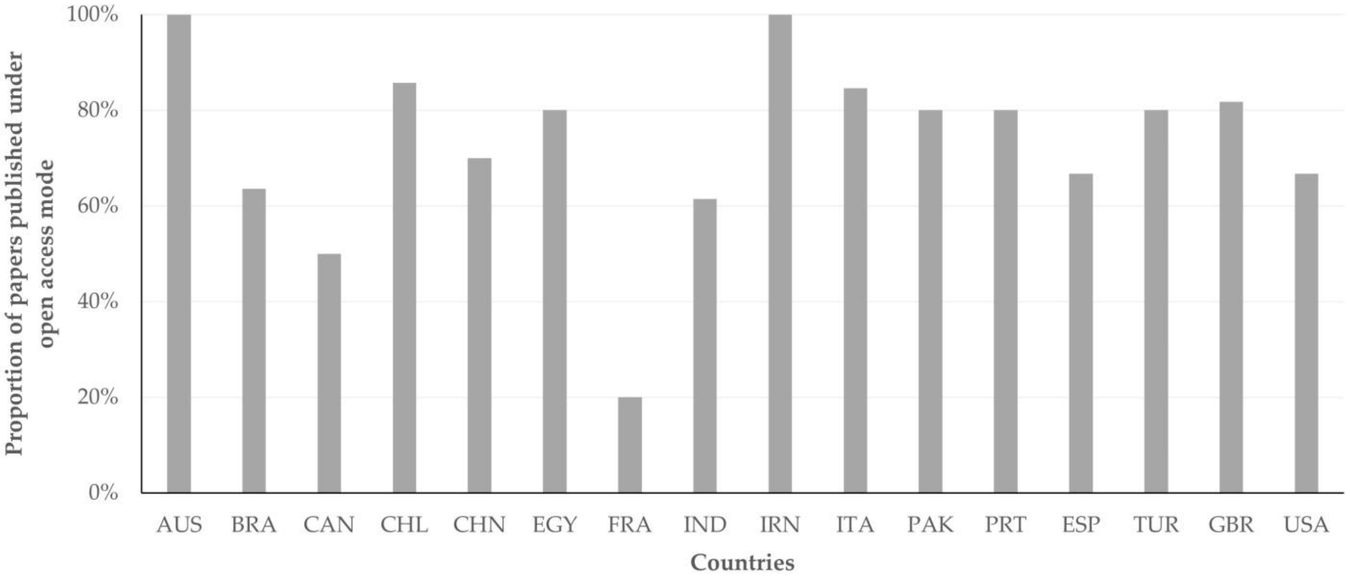

[1] Abbreviations of country names according to the International Naming Convention ISO 3166 [International Organization for Standardization 2024].

Table S22. Results of univariable analyses for associations with the number of yearly citations received per paper on veterinary or animal studies scientometrics.

Parameter (n = 11)	r_{sp}	p value
Year of publication of paper	–0.115	0.14
International collaborations in origin of paper	0.133	0.09
Number of authors in published paper	0.105	0.18
Number of cited references in published paper	0.283	0.0003
Number of databases used for record search	–0.019	0.81
Timespan covered in the search for record search	0.247	0.002
Number of records obtained	0.073	0.36
Number of listed keywords in published paper	0.138	0.08
Inclusion of specific animal species in searches for record search	0.165	0.035
Topic of assessment	0.061	0.44
Accessibility of published paper	0.200	0.010

Table S23. The median number of yearly citations per published paper on veterinary or animal studies scientometrics from the 16 countries with most papers (≥ 5).

Country	Median (interquartile range) of citations per published paper yearly
Australia	2.0 (5.2)
Brazil	1.0 (1.9)
Canada	1.9 (2.9)
Chile	0.0 (0.7)
China	3.5 (3.5)
Egypt	2.0 (2.5)
France	0.2 (1.0)
India	0.5 (2.0)
Iran	1.8 (1.3)
Italy	2.0 (4.8)
Pakistan	4.7 (1.2)
Portugal	5.7 (6.3)
Spain	2.3 (3.9)
Turkiye	2.0 (1.7)
United Kingdom	2.0 (6.6)
United States of America	1.7 (1.1)

Figure S7. Box and whisker plot of the yearly citations per published paper on veterinary or animal studies scientometrics in accord with the topic of study and scientometric assessment (only most frequently assessed topics shown).

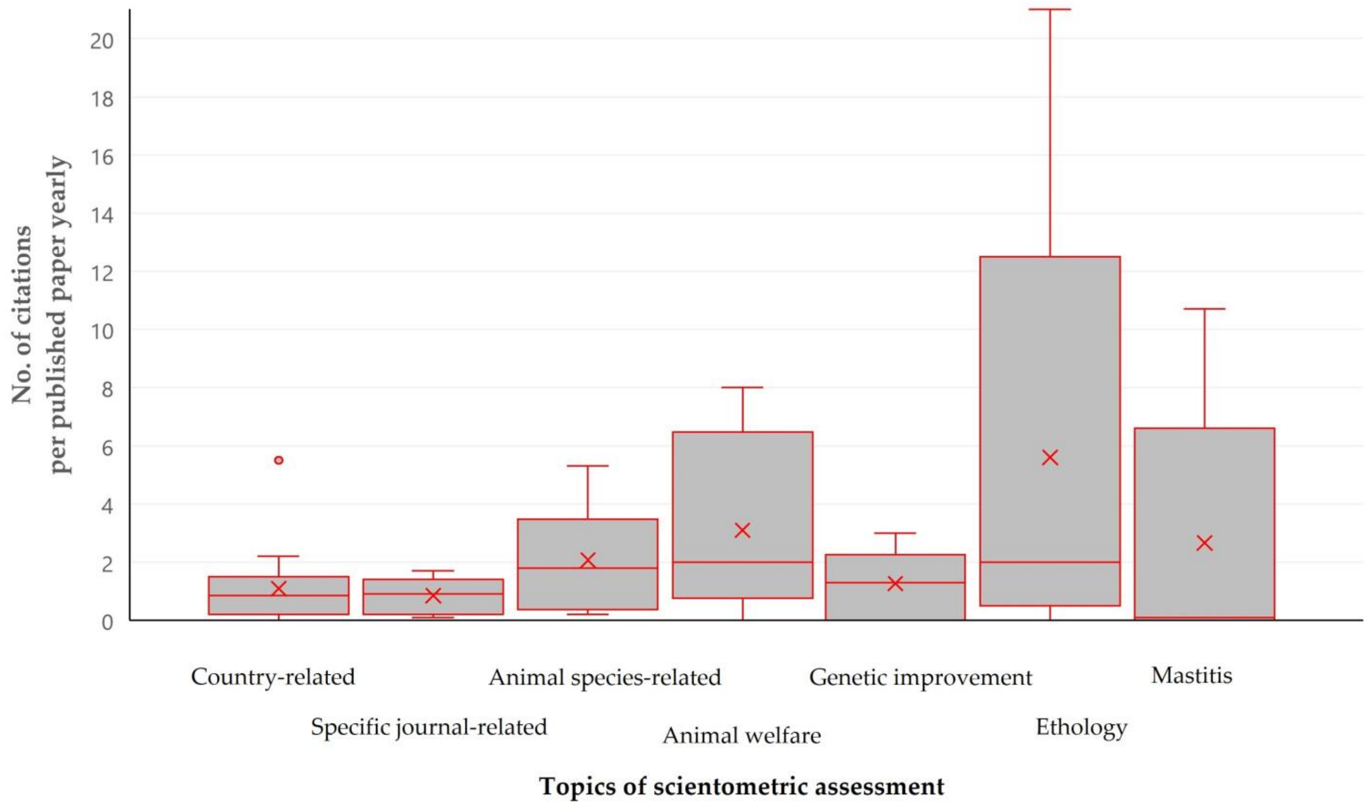

Figure S8. Scree-plot of results of principal components analysis for the yearly citations received by the published papers on veterinary or animal studies scientometrics.

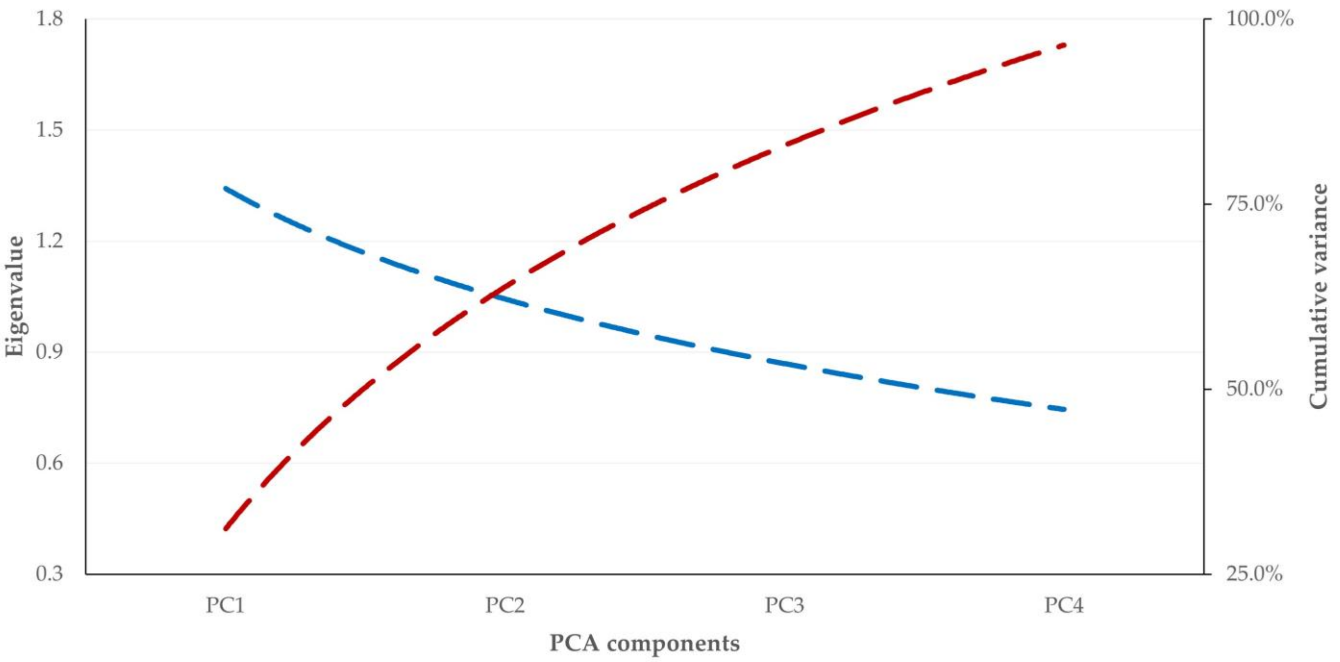

Supplement: Supplementary file 1 [file animals-14-03132-s001.zip › animals-3262794-supplementary.pdf]
